# Comparative Evaluation of EfficientNet-B3, Vision Transformers, and RETFound for Seven-Category Retinopathy Fundus Image Classification

**DOI:** 10.3390/diagnostics16193109

**Published:** 2026-09-24

**Authors:** Majid Alutaibi, Meshari Alazmi

**Affiliations:** College of Computer Science and Engineering, University of Ha’il, Ha’il 81411, Saudi Arabia

**Keywords:** retinal disease classification, deep learning, EfficientNet-B3, fundus photography, foundation model, cross-validation

## Abstract

**Background/Objectives:** Retinal fundus photography is the most widely available means of screening for sight-threatening disease, but it is difficult to read: several conditions leave overlapping signs on the same photograph, the features that separate them are often subtle and confined to small regions of the retina, and a single image carries no systemic context, so an automated reader must resolve categories on which experienced clinicians themselves agree only moderately. **Methods:** We compared five deep learning architectures—EfficientNet-B3, DenseNet-121, ViT-Base/16, Inception-V3, and RETFound ViT-Large—for simultaneous seven-class retinal disease classification on a multi-source fundus image benchmark, in a retrospective computational study using stratified five-fold cross-validation. We pooled 11,223 patient-eye fundus images from three publicly available, de-identified datasets (ODIR-5K, RFMiD, and APTOS 2019) spanning age-related macular degeneration (AMD), cataract, diabetic retinopathy (DR), glaucoma, hypertensive retinopathy, pathological myopia, and normal fundus; the cohort was markedly class-imbalanced (DR, 62.5%; normal, 25.6%; each remaining class < 3%), and no new patient data were collected. Images were deduplicated by MD5 hash and split at the patient level to eliminate data leakage, with a fixed 15% held-out test set and five stratified cross-validation folds; training images received Ben Graham illumination normalization and class-balancing augmentation, while validation and test images were unaugmented. Three ImageNet-pretrained CNNs (EfficientNet-B3, DenseNet-121, Inception-V3), ViT-Base/16, and RETFound ViT-Large (pretrained on 1.6 million retinal photographs via masked autoencoding) were trained; the primary outcome was the five-fold mean weighted-averaged F1 score, pairwise comparisons used paired *t*-tests on fold-level F1 (df= 4), and Grad-CAM was applied for explainability. **Results:** EfficientNet-B3 achieved the highest five-fold mean accuracy (95.53%; weighted-F1, 0.9507) and significantly outperformed RETFound ViT-Large (ΔF1=+0.0255; t=9.70; p<0.001), ViT-Base/16 (ΔF1=+0.0075; t=9.20; p<0.001), and Inception-V3 (ΔF1=+0.0103; t=8.38; p<0.001), while performing comparably to DenseNet-121 (t=2.24; p=0.089); Grad-CAM showed clinically plausible activation localization for the CNN architectures. **Conclusions:** A compact, resource-efficient CNN thus matched or exceeded a substantially larger retinal foundation model and Vision Transformers, indicating that larger domain-specific foundation models are not inherently superior for heterogeneous retinal screening; because such compact models run on commodity hardware, they may offer more practical screening solutions for community, rural, and resource-limited settings where ophthalmologist coverage is scarcest. External validity across imaging sources and populations remains unproven, underscoring that prospective, multicenter validation must precede clinical deployment. A provenance audit of this pooled cohort subsequently found it confounded. The audit identified five failure modes, three of them decisive: image width alone reproduced the diabetic retinopathy label at 99.98% accuracy using no retinal pixels, source and label were almost perfectly dependent with a Cramér’s V of 0.9996, and diabetic retinopathy recall was exactly 1.0000 for all five architectures. The cohort was rebuilt from a single source with per-eye labels, and nine architectures were retrained under one protocol with external validation. The corrected headline is a macro-AUC of 0.930 internally and 0.978 externally, with a macro-F1 of 0.686. No architecture is declared superior, and the pooled-cohort conclusion does not stand.

## 1. Introduction

Retinal diseases collectively represent the leading preventable causes of irreversible visual impairment and blindness worldwide. The World Health Organization estimates that at least 2.2 billion people globally suffer from a vision impairment, with conditions including diabetic retinopathy (DR), glaucoma, age-related macular degeneration (AMD), and cataract responsible for the majority of preventable vision loss [1]. Early detection through retinal fundus photography offers an established, cost-effective screening pathway, but global access to ophthalmologic expertise remains severely limited, particularly in low- and middle-income countries.

Automated image-based classification systems have demonstrated substantial promise in addressing this gap. Landmark studies established that deep convolutional neural networks (CNNs) could detect diabetic retinopathy at or above specialist-level performance from fundus photographs [2,3]. Subsequent work expanded automated screening to glaucoma, AMD, and multi-disease settings [4,5,6,7]. Despite these advances, most published systems address single diseases on single-institution datasets, limiting their utility for real-world deployment where multiple co-occurring conditions must be differentiated simultaneously across diverse imaging environments.

Beyond these landmark studies, deep learning has been applied to fundus photography across most major retinal conditions, as reviewed for ophthalmology generally [8] and for fundus images specifically [9]. Deep networks have been trained to identify diabetic retinopathy [10], glaucomatous optic neuropathy [7], and age-related macular degeneration, both from individual images [11] and as patient-level severity [12]. Multi-disease systems have extended this to dozens of fundus diseases and conditions within a single model [13], and fundus photographs have been shown to carry systemic information such as cardiovascular risk factors [14]. Two features of this literature bear directly on the present study. First, hypertensive retinopathy is defined by signs that overlap with other vascular retinopathies and is interpreted alongside blood pressure rather than from the image alone [15], which limits what an image-only label can represent. Second, model performance depends on the reference standard used to label the images: adjudicated grading changes both the measured accuracy of models and the conclusions drawn from them [16].

Vision Transformers (ViTs) and, more recently, large foundation models pretrained on domain-specific data represent the current frontier in medical image classification [17,18]. RETFound, a self-supervised ViT-Large pretrained on 1.6 million retinal fundus photographs via masked autoencoding, has been proposed as a general-purpose retinal AI platform capable of outperforming supervised baselines on multiple retinal tasks [19]. However, whether this advantage extends to simultaneous multi-class, multi-source classification—the scenario most relevant to real-world screening—has not been rigorously evaluated against compact CNNs under controlled experimental conditions.

Reported performance is only as meaningful as the evaluation behind it. Systematic reviews comparing deep learning with health-care professionals found that few studies validated their models externally, that reporting was often incomplete [20], and that many claims of expert-level performance rested on designs at high risk of bias [21]. A recurring failure is shortcut learning, in which a model exploits features that correlate with the label in the training data but not with the disease [22]. Chest radiograph models have detected pneumonia partly by recognizing the hospital system that acquired the image [23], COVID-19 classifiers have selected dataset-specific shortcuts over pathological signal [24], and hip-fracture models have relied on confounding patient and healthcare variables [25]. A closely related failure is data leakage, identified as a widespread source of irreproducible results across machine learning-based science [26]. In medical imaging, splitting at the level of the image or slice rather than the subject has inflated test accuracy for brain MRI [27] and for optical coherence tomography [28]. Broader reviews have documented how dataset composition and evaluation choices distort results in medical imaging research [29], to the point that methodological flaws and underlying biases left none of the COVID-19 imaging models examined in one large review suitable for clinical use [30]. Reporting guidelines such as CLAIM [31] and TRIPOD+AI [32] were developed in part to make these problems visible.

We conducted a systematic, head-to-head comparison of five architectures, including both ImageNet-pretrained CNNs and RETFound ViT-Large, for seven-class simultaneous retinal disease classification on a large multi-source fundus image benchmark. We further evaluated model explainability using gradient-weighted class activation mapping (Grad-CAM). We examined whether domain-specific retinal pretraining confers a classification advantage over compact, general-purpose networks in this multi-source screening setting.

That literature shaped the present analysis directly. We audited the pooled cohort for exactly the source-driven shortcuts and leakage described above, found them, and rebuilt the study on a single-source cohort. The pooled-cohort analysis and the corrected analysis are both reported, so that a reader can see which design choices moved which results.

## 2. Methods

Structure of this section: Section 2.1, Section 2.2, Section 2.3, Section 2.4, Section 2.5, Section 2.6, Section 2.7, Section 2.8, Section 2.9 and Section 2.10 describe the pooled cohort and the analysis performed on it. Section 2.3 sets out the provenance, licensing and ethical status of the three source datasets. Section 2.11, Section 2.12, Section 2.13 and Section 2.14 describe the provenance audit of that cohort, how the cohort was rebuilt once the audit failed, the training protocol applied to the rebuilt cohort, and the statistical analysis of the corrected results. Both analyses are reported throughout, the pooled one alongside the corrected one.

### 2.1. Study Design and Ethical Considerations

We conducted a retrospective computational study using three publicly available, de-identified retinal fundus image datasets. No new patient data were collected. All source datasets are distributed in de-identified form. Their individual license terms differ and are set out in Section 2.3. Institutional review board (IRB) approval was not required. This study adheres to the Checklist for Artificial Intelligence in Medical Imaging (CLAIM) [31].

### 2.2. Datasets and Disease Categories

We pooled images from three publicly available fundus image datasets: the Ocular Disease Intelligent Recognition dataset (ODIR-5K) [33], the Retinal Fundus Multi-Disease Image Dataset (RFMiD) [34], and the APTOS 2019 Blindness Detection dataset [35]. We retained images belonging to seven mutually exclusive disease categories: AMD, cataract, DR, glaucoma, hypertensive retinopathy, pathological myopia, and normal fundus. After pooling and the leakage-control procedure described below (deduplication and patient-level assignment), the analytic cohort comprised 11,223 unique patient-eye images. Reflecting the epidemiology of the source datasets, the cohort was markedly class-imbalanced: DR (62.5%) and normal fundus (25.6%) together accounted for 88% of images, while each of the five remaining categories represented fewer than 3% (Table 1). This imbalance—rather than an artificially balanced benchmark—reflects the realistic screening distribution and motivated our class-balancing augmentation strategy. Dataset characteristics are summarized in Table 1.

### 2.3. Dataset Provenance, Licensing and Ethical Status

Figure 1 shows one representative image from each class in both datasets, after the harmonization described below.

Table 2 records, for each public dataset, the access route, the originating data source, the annotation procedure, the de-identification status and the license under which it is used. Three points require comment rather than a table entry.

License terms are not uniform across the three datasets, and we do not describe them as if they were: RFMiD is published under the Creative Commons Attribution 4.0 International license, which permits reuse and redistribution with attribution. ODIR-5K carries no equivalent public license: access is granted on registration at the dataset’s official page and is governed by the terms accepted there, which permit research use but do not grant redistribution rights. APTOS 2019 is governed by the competition rules accepted on the hosting platform. Accordingly, this study redistributes no images from any source. What is released is code, the label-mapping rules, the per-image manifest schema, the split assignment and the per-sample prediction arrays, none of which contains image data.

Expert validation is a property of the source datasets, not of this study: ODIR-5K labels were assigned by trained human readers under quality control by the data provider, with one read per eye and no adjudication reported; RFMiD labels were assigned by adjudicated consensus of two senior retinal experts. No image was re-graded for the present work, and no ophthalmologist on this author team reviewed the source annotations. Where the reliability of those annotations bears on our results, we say so: ODIR-5K provides a single read per eye with no adjudication and no ancillary testing, which is stated in the Limitations as a bound on the attainable ceiling.

Anonymization was performed by the data providers before release: ODIR-5K is distributed with patient-identifying information removed, and its publication follows the ethical and privacy rules of the jurisdiction in which it was collected. RFMiD is distributed de-identified by its publishing institutions. This study received no identifiable data, collected no new patient data, and performed no re-identification of any kind. Patient identifiers used here are dataset-internal indices supplied with the source distributions, not clinical record numbers.

### 2.4. Image Preprocessing

We applied Ben Graham illumination normalization to all images before training and inference [36]. This method subtracts a Gaussian-blurred version of each image (σ=radius×0.06) from the original, effectively removing slow-varying illumination gradients arising from differing camera hardware, lens characteristics, and acquisition conditions across the three source datasets. We resized all images to each model’s native input resolution (299 × 299 for Inception-V3, 300 × 300 for EfficientNet-B3, 224 × 224 for all others) and applied standard ImageNet channel normalization.

In a preliminary single-split experiment, performance degradation under CLAHE preprocessing ranged from 5.9 percentage points (Inception-V3) to 16.9 percentage points (DenseNet-121), confirming that Ben Graham normalization is essential for this multi-source benchmark.

### 2.5. Data Partitioning and Augmentation

Leakage control: An initial audit of the pooled data revealed severe data leakage introduced by an earlier pipeline that had applied offline augmentation *before* splitting: 64.6% of test patients also appeared in the training set as differently augmented copies, and 49 images were content-identical across splits. To eliminate this, we (i) pooled all images into a single registry, (ii) extracted a canonical patient-eye identifier from each filename, (iii) removed content-identical images by MD5 hash, and (iv) performed all subsequent splitting at the patient level. We verified zero MD5 overlap between any two splits before training.

Near-duplicate detection: MD5 hashing detects byte-identical files only, which is the least sensitive form of the check required for a pooled collection. For the reconstructed cohort, we therefore added a perceptual sweep. Two 64-bit hashes were computed per image, a horizontal-gradient hash and a mean-threshold hash, and a pair was a candidate only if both agreed within four bits; candidates were confirmed by Pearson correlation at 128×128 on the masked fundus interior, with a threshold of 0.98. Masking matters: an earlier attempt at 64×64 without a mask and with a threshold of 0.95 returned tens of thousands of matches including normal–DR pairs, because every fundus photograph is a bright disc on a dark circular field and that shared geometry dominates the statistic at low resolution. The detector was validated before use: it recovered 40 of 40 identical images and 40 of 40 copies re-encoded as JPEG at quality 70, and produced no false positive in 4000 random unrelated pairs.

Partitioning: We reserved 15% of patients (stratified by class) as a fixed held-out test set comprising 1679 images (exactly one image per patient). The remaining patients were partitioned into five class-stratified cross-validation folds. For each fold *k*, the four other folds formed the training set and fold *k* served as the validation set (one image per patient; 1906–1913 validation images per fold).

Augmentation: Because the cohort was severely imbalanced, we applied class-balancing offline augmentation to training images only—random horizontal and vertical flips, up to 15° rotation, and color jitter (brightness, contrast, saturation ±0.2)—oversampling minority classes toward parity and yielding approximately 35,300 training images per fold. Validation and test images were never augmented, ensuring that all reported performance is measured on unaugmented, patient-disjoint images.

### 2.6. Model Architectures

We evaluated five deep learning architectures (Table S1). Three were compact ImageNet-pretrained CNNs: EfficientNet-B3 (12 million parameters; compound scaling) [37], DenseNet-121 (8 million parameters; dense skip connections) [38], and Inception-V3 (23 million parameters; multi-scale parallel pathways) [39]. One was an ImageNet-pretrained Vision Transformer: ViT-Base/16 (86 million parameters) [17]. The fifth was RETFound ViT-Large (307 million parameters), a self-supervised foundation model pretrained on 1.6 million retinal fundus photographs via masked autoencoding with 75% patch masking, fine-tuned using the authors’ published protocol [19]. All classification heads were replaced with fully connected layers mapping to seven output classes.

### 2.7. Training Protocol

We trained all models using the AdamW optimizer with cosine annealing learning rate scheduling. CNN architectures used an initial learning rate of $1\times 10^{-4}$, weight decay of $1\times 10^{-4}$, batch size of 32, and 50 training epochs with early stopping on validation weighted-F1 (patience = 10 epochs). ViT-Base/16 used an initial learning rate of $5\times 10^{-5}$, weight decay of $1\times 10^{-2}$, batch size of 16, and 30 epochs. RETFound ViT-Large used the authors’ fine-tuning protocol: initial learning rate $5\times 10^{-4}$, layer-wise learning rate decay (factor 0.75), batch size of 16, and 50 epochs. We applied categorical cross-entropy loss without class weighting. All experiments were conducted on a single NVIDIA A100 GPU.

### 2.8. Evaluation Metrics

The primary outcome was the five-fold mean support-weighted F1 score (weighted-F1), computed as the mean of the seven per-class F1 scores weighted by class support. We also report overall accuracy as a secondary metric. Because weighted-F1 is influenced by the majority DR and normal classes under this imbalance, it should be interpreted as an overall-performance summary; macro-averaged F1 (equal weighting of all classes) would provide a more stringent, minority-class-sensitive complement and is a recommended target for future evaluation. We report mean and sample standard deviation (SD; denominator n−1=4) across five folds.

### 2.9. Statistical Analysis

We assessed pairwise differences in fold-level weighted-F1 using paired *t*-tests (two-tailed, df=4, α=0.05) with sample SD (denominator n−1=4) throughout [40]. For each comparison we additionally report the 95% confidence interval of the mean paired difference and the standardized paired effect size (Cohen’s $d_{z}$). Had Holm–Bonferroni correction [41] been applied across the four pairwise comparisons, all three statistically significant results (t≥8.38) would remain significant at adjusted thresholds of 0.017, 0.025, and 0.05, and the DenseNet-121 comparison (p=0.089) would remain non-significant.

### 2.10. Explainability Analysis

We applied Grad-CAM [42] to all five architectures using each model’s best-performing fold checkpoint. For ViT-Base/16 and RETFound, we applied Grad-CAM to the final transformer block with a spatial reshape transform. We applied t-SNE to penultimate-layer features from Inception-V3 (fold 4; N=1906; perplexity 40) to visualize the learned disease-class feature space.

### 2.11. Provenance Audit of the Pooled Cohort

We audited the provenance of the pooled cohort of Section 2.2. The audit consisted of four probes, P1–P4, computed from an image manifest and from the images themselves; together they formed a gate that had to be passed before any model was trained. Two of the four were exact enumerations and two were not. We say which is which rather than present all four as measurements of the same kind. Values on the pooled cohort and on the rebuilt cohort appear in the Results.

P1—Per-eye label independence: For every patient contributing two fundus images, the left and right labels were compared; the probe reports the proportion of two-eye patients whose eyes carried different labels. Passing required a discordance of at least 5%. A discordance of 0% was not unusually symmetric disease. It was a cohort whose labels were not eye-level labels at all, since unilateral and asymmetric presentation was routine in all seven categories. P1 enumerated every eye in the cohort.

P2—Resolution uniformity: The stored pixel width was read from the file header; passing required exactly one distinct width. As a complementary diagnostic we recorded the accuracy of the best single-threshold rule on width alone, which used no retinal content. As run on the rebuilt cohort, the probe read the stored width of the first 400 images in manifest order rather than of all 5911, so its verdict was a sample statement and not an enumeration. It is cheap to run exhaustively and should be. Re-running it over all 5911 widths is one of the two changes we would make to the gate.

P3—Color and field-of-view recoverability: Twenty-two anatomy-free scalars were computed per image on a 64×64 downsampled version: per-channel means, standard deviations and 5th, 50th and 95th percentiles; the mean and standard deviation of saturation; the mean of the per-pixel channel maximum; the non-black pixel fraction; and the three pairwise channel differences. A random forest of 300 trees was scored by 5-fold stratified cross-validation with balanced accuracy, first for acquisition source and then for class; the class probe passed if balanced accuracy was no greater than chance plus 0.15. This probe estimated, where P1 and P4 enumerated. On the rebuilt cohort, it was fitted to a random subsample of 2200 of the 5911 eyes, with subsample generator seed 0 and random state 0 for both the forest and the fold assignment. Its mean cross-validated balanced accuracy was a single point estimate. The sampling uncertainty that came with the subsampled draw and the fold split was not quantified, and no interval is attached to it anywhere in this paper. Attaching a resampling interval to P3 is the second change we would make to the gate.

P4—Source composition: Distinct acquisition sources contributing to the training cohort were counted; passing required exactly one. P4, like P1, enumerated every eye. It measured no leakage. It removed the variable through which source-to-label leakage would have had to travel.

### 2.12. Cohort Reconstruction

The internal cohort of the reconstructed analysis was ODIR-5K [33] alone. That foreclosed cross-source label leakage *by construction* rather than reducing it by measurement: with one acquisition source, there was no provenance variable through which source could predict label. Leakage above the majority-class baseline was +0.00 percentage points, and Cramér’s *V* between source and label was 0.0000 on the held-out test set. The cost was that the internal test set measured one acquisition protocol in one population and is therefore not described as external validation.

Labels were derived per eye. ODIR-5K distributes one row per *patient*, carrying per-patient N/D/G/C/A/H/M/O indicator columns alongside two per-*eye* free-text columns, Left-Diagnostic Keywords and Right-Diagnostic Keywords. Applying the patient-level columns to both eyes is the natural reading of the file, and it produces a left/right label discordance of exactly 0%. Parsing each eye’s own keyword column gives 17.1% (444 of 2603 two-eye patients). We therefore derived each eye’s label from its own keyword column by a fixed, deterministic keyword-to-class map, dropping eyes that mapped to more than one of the seven classes and eyes that mapped to none, so the classes stayed mutually exclusive. Of 7000 eyes, 5911 (84.4%) were retained; 184 were dropped as multi-class within the taxonomy and 905 as outside it. The parser was a *function* on its domain, and we established that exhaustively: the retained labels arose from 155 distinct keyword strings, none of which mapped to more than one class, so the label derivation for all 5911 cohort eyes could be checked by reading 155 rows without opening a single image.

Harmonization cropped every image to the bounding box of the illuminated fundus before resizing and re-encoding, so field-of-view differences were removed rather than carried into the model input. Partitioning was at the patient level. Patients, not images and not eyes, were assigned by fixed random seed either to a held-out test set or to one of five cross-validation folds; both eyes of a patient therefore fell on the same side of every split, and fellow-eye leakage was not biologically plausible. The held-out test set contained 872 images from 495 patients, and the remaining patients formed the five folds. Within each fold the validation set was split patient-wise again into an early-stopping half and a disjoint reporting half, so epoch selection touched only the first and the reported validation metric only the second.

RFMiD [34] passed through the identical harmonization into a separate directory that no training code read: 721 images, 4 of the 7 classes present. No threshold was refitted on it, no external example informed any selection decision, and no external claim is made for the classes it does not cover.

Nine architectures were trained under a single protocol: the five of the original submission, plus ConvNeXt-Small, ConvNeXt-Base, EfficientNetV2-S and Swin-Base. One protocol for all nine is what makes the observed spread an architecture effect and not a tuning effect. RETFound [19] was retrained with a repaired position-embedding load. The widely copied fine-tuning pattern admits a checkpoint tensor only when its key is present in the encoder state dictionary *and* the two shapes are equal. The published checkpoint was pretrained at 224 px, so its position-embedding tensor has shape (1197,1024): one class token plus a 14×14 patch grid. A ViT-Large fine-tuned at 384 px requires (1577,1024), one class token plus a 24×24 grid. The shape guard rejected the tensor, the state dictionary loaded non-strictly, and the model trained with randomly initialized position embeddings while every log line reported a successful load. We interpolated the position embedding bicubically from the 14×14 to the 24×24 grid, printed the shape change, and raised an error if fewer than 100 encoder tensors matched. The RETFound arm of the reconstructed analysis was trained with the repair in place.

### 2.13. Training Protocol for the Reconstructed Analysis

All nine architectures were trained under one protocol; only the input resolution and the micro-batch size differed, and those followed from each backbone’s native input and from fitting the model on one GPU. Optimization used AdamW ($\beta_{1}=0.9$, $\beta_{2}=0.999$, weight decay 0.05) with discriminative learning rates: $3\times 10^{-4}$ for the classifier head and $1\times 10^{-4}$ for the backbone on the ImageNet-pretrained models, and $1\times 10^{-3}$/$5\times 10^{-4}$ for RETFound, the latter being the official RETFound base learning rate of $5\times 10^{-3}$ scaled to an effective batch size of 32. The transformer families additionally used layer-wise learning-rate decay (0.65 for RETFound, 0.75 for ViT-Base/16, 0.8 for Swin-Base). The schedule was cosine with three warm-up epochs and a period matched to the number of epochs actually run rather than to the nominal cap, since a period set for a cap the run never reaches truncates the decay. Training ran for at most 30 epochs with early stopping on validation macro-AUC, a patience of 12, evaluated on the early-stopping half of the validation split only. Loss was cross-entropy with label smoothing at 0.05. An exponential moving average of the weights (decay of 0.9998) was maintained and was what was evaluated and checkpointed. Gradient accumulation brought every effective batch to 24–36 images. Mixed precision was bfloat16. RandAugment, mixup and CutMix were deliberately absent: each was measured or judged to harm the rare classes on a cohort of this size. Each fold trained on 4024 images. Runs reported here used seed 42. Per-architecture settings are given in Table 3.

Cohort flow: ODIR-5K contributed 7000 eyes. The single-label filter retained 5911 (84.4%), dropped 184 (2.6%) carrying two or more of the seven classes, and dropped 905 (12.9%) whose findings lay outside the taxonomy. Of the 5911 retained eyes, 872 from 495 patients formed the held-out test set and 5039 entered five patient-disjoint cross-validation folds, each training on 4024 images. RFMiD contributed 721 images, used only for external evaluation.

Software: Python 3.11.5, PyTorch 2.11.0 with CUDA 12.8, timm 1.0.25, scikit-learn 1.8.0, NumPy 2.3.5 and Pillow 12.0.0, on a single GPU.

Status of the analysis plan: The analysis plan, including the primary endpoint, the single pre-specified pairwise contrast and every uncertainty procedure, was fixed in writing before the held-out test set was scored. It was not deposited in a public registry, and this study carries no registration number. We therefore describe the plan as pre-specified rather than pre-registered, and a reader should weight it accordingly: the ordering is documented in our repository history, not certified by a third party.

### 2.14. Revised Statistical Analysis

The paired *t*-tests of Section 2.9 were computed on five cross-validation folds with df=4 and treated the folds as independent observations. They were not independent. Five-fold training sets shared 75% of their examples, fold differences were positively correlated, and treating them as independent was anti-conservative. The re-analysis applied the Nadeau–Bengio [43] correction for the resampled *t* statistic: its variance multiplier $1/k+n_{2}/n_{1}=0.455$ replaced the naive 0.200, which gave a corrected standard error 1.51× the naive one and an interval of the fold mean ±1.87× SD with $t_{4,0.975}=2.776$.

The primary endpoint of the re-analysis was one-versus-rest macro-averaged AUC on the held-out test set. Intervals and paired contrasts came from a patient-level paired bootstrap with B=2000 replicates, percentile limits and seed 20260831. Patients were resampled rather than images: a patient’s two eyes shared systemic disease status, media opacity and acquisition conditions. Every paired contrast drew on a *single shared resampling index matrix* across all models and metrics, so each paired difference came from the same bootstrap world. Holm–Bonferroni correction was applied within each pre-declared family, defined by metric and containing all 36 architecture pairs. Paired accuracy used McNemar’s test [44]. No accuracy comparison was reported for a contrast with fewer than 30 discordant pairs. As a pre-specified sensitivity check, the headline intervals were recomputed with a class-stratified bias-corrected and accelerated (BCa) bootstrap [45] with B=10,000 replicates; they agreed with the percentile intervals to within 0.003.

One deviation from the analysis plan is disclosed: DeLong’s test [46] was pre-specified for the macro-AUC contrasts. Its variance estimator assumes independent observations, and 754 of the 872 held-out test images (86.5%) shared a patient with another test image, so DeLong would understate the variance of every contrast on exactly the axis that matters here. The macro-AUC and macro-F1 contrasts therefore used the paired patient-level bootstrap, on the same shared index matrix as every other interval. This substitution widens every interval and is therefore conservative with respect to the reported contrasts.

Rather than assert uniform interval coverage, we state which results carry an interval and which do not. Intervals accompany the held-out internal test metrics of each architecture and of the ensemble, the ensemble’s per-class F1 values, the ensemble’s external metrics on RFMiD, and every pairwise between-architecture contrast. Four sets of results are printed as bare point estimates, and none supports a comparative claim: the cross-validation summaries, given as fold means with standard deviations and the Nadeau–Bengio multiplier rather than as resampling intervals; the per-architecture external results, on which no comparison or selection is made; the post hoc calibration summaries; and the post hoc per-class operating characteristics, sensitivities and specificities at the arg-max decision rule included. No exact binomial (Clopper–Pearson [47]) interval is computed anywhere in this paper, for recall and specificity or for anything else.

The minimum detectable paired difference on this test set was about 0.034 macro-F1 and about 2.1 percentage points of accuracy, at 80% power, α=0.05 and an assumed inter-model correlation of ρ=0.7. The re-analysis is therefore declared in advance to be powered to *characterize* architectures, not to *rank* them. It cannot separate architectures that differ by less than that, and where the 95% confidence interval of a between-architecture difference contains zero, we claim no winner: not in the abstract, not in a figure caption, and not through ordering or emphasis.

## 3. Results

### 3.1. Five-Fold Classification Performance

Table 4 presents five-fold cross-validation performance for all five architectures. EfficientNet-B3 achieved the highest five-fold mean accuracy (95.53%; weighted-F1, 0.9507; SD, 0.0029), with fold-level F1 ranging narrowly from 0.9469 to 0.9527, demonstrating consistent convergence across all five partitions. The performance ordering by weighted-F1 was consistent across all five folds: EfficientNet-B3 > DenseNet-121 > ViT-Base/16 > Inception-V3 ≫ RETFound ViT-Large.

DenseNet-121 ranked second (mean accuracy, 94.88%; weighted-F1, 0.9448) but exhibited greater cross-fold variability (SD, 0.0058). ViT-Base/16 and Inception-V3 performed comparably (weighted-F1, 0.9432 and 0.9404, respectively), both consistently exceeding RETFound across all five folds. RETFound ViT-Large achieved the lowest in-distribution performance (mean accuracy, 93.74%; weighted-F1, 0.9251; SD, 0.0076) despite pretraining on 1.6 million retinal fundus photographs. Notably, RETFound’s highest single-fold F1 (0.9319, fold 4) remained 1.5 percentage points below EfficientNet-B3’s lowest single-fold F1 (0.9469, fold 0), confirming that the performance ordering was not an artifact of a single fold. EfficientNet-B3’s 2.6-percentage-point advantage over RETFound (weighted-F1, 0.9507 vs. 0.9251) was achieved with a substantially smaller and more computationally efficient model.

One-versus-rest AUC-ROC curves (Figure S1) were consistent with the weighted-F1 ranking. Hypertensive retinopathy showed the lowest AUC across all models, reflecting diagnostic overlap with glaucoma and AMD.

### 3.2. Statistical Comparisons

Paired *t*-tests confirmed statistical significance of all pairwise comparisons except EfficientNet-B3 vs. DenseNet-121 (Table S2). EfficientNet-B3’s advantage over RETFound was highly consistent across all five folds (per-fold ΔF1: +0.0347, +0.0204, +0.0277, +0.0241, +0.0208; t=9.70; p<0.001). The advantage over ViT-Base/16 (t=9.20; p<0.001) and Inception-V3 (t=8.38; p<0.001) was equally consistent. The EfficientNet-B3 vs. DenseNet-121 comparison showed a positive direction but did not reach statistical significance (t=2.24; p=0.089); these two architectures are considered comparably performing.

### 3.3. Explainability

Grad-CAM activation maps (Figure S2) demonstrated that CNN architectures produced activation patterns focused on pathologically relevant retinal regions—exudates and neovascularization for DR, optic disc rim thinning for glaucoma, drusen deposits for AMD, and arteriolar caliber changes for hypertensive retinopathy—consistent with established ophthalmoscopic diagnostic criteria. ViT-Base/16 and RETFound produced more diffuse, patch-level attention maps, with RETFound showing the least spatially focused activations.

The dominant misclassification boundary occurred between hypertensive retinopathy and glaucoma, and between AMD and hypertensive retinopathy—conditions sharing overlapping funduscopic features including optic disc structural changes, peripapillary atrophy, and posterior pole vascular abnormalities. t-SNE visualization confirmed that all seven classes formed compact, largely separable clusters, with partial overlap exclusively at the hypertensive retinopathy/glaucoma/AMD boundary.

### 3.4. Classical Machine Learning Reference

Four classical classifiers trained on EfficientNet-B3 penultimate-layer features achieved competitive performance: a support vector machine (radial basis function kernel with principal-component reduction; accuracy, 93.5%; weighted-F1, 0.935), LightGBM (accuracy, 91.7%; weighted-F1, 0.917), XGBoost (accuracy, 90.0%; weighted-F1, 0.899), and random forest (accuracy, 85.8%; weighted-F1, 0.855). The support-vector-machine pipeline performed within 2.0 percentage points of EfficientNet-B3 end-to-end while enabling CPU-based inference, indicating that the learned feature representation is highly separable.

### 3.5. Provenance Audit: Findings

The results reported above are those of the pooled cohort. A provenance audit of that cohort found it confounded. Every diabetic retinopathy (DR) image entered the pool from a 1024 px corpus and every image of the six other classes from 512 px corpora, so acquisition source and diagnostic label were very nearly the same variable. This subsection reports what the audit found; the two that follow report the rebuilt cohort and set the two sets of estimates side by side. The pooled-cohort results above are not overwritten here. They are contrasted.

Failure mode 1: the label is recoverable from the file header: DR images were stored at a native width of 1024 px and all six other classes at 512 px. The rule width == 1024 → DR assigned the correct class to 11,221 of the 11,223 images. That is 99.98% accuracy from zero retinal pixels. Cramér’s *V* between acquisition source and class label was 0.9996, against zero for independence, and DR recall in the original analysis was exactly 1.0000 for every one of the five architectures. A recall of exactly 1.0000 reproduced across five networks of different inductive bias—convolutional, inception-style, transformer, retinal foundation model—is not a property those networks share. It indicates a label recoverable from file metadata alone, and every architecture recovered it.

Failure mode 2: the color signature survives harmonization: Uniform re-rendering to 512×512 does not remove the confound. With every image re-rendered at that size, 22 color and field-of-view scalars containing no anatomical information still recovered acquisition source at 96.95% balanced accuracy against a chance level of 33.3%, and class at 38.1% against a chance level of 14.3%. Resizing removes the header cue and leaves the corpus-level color and framing signature intact. That is why the correction had to be structural rather than a change of preprocessing.

Failure mode 3: per-patient labels stamped onto both eyes: ODIR-5K distributes eight patient-level diagnosis columns (N/D/G/C/A/H/M/O). Applying those columns to both eyes of a patient, as the pooled analysis did, yields a left/right label discordance of 0% across 1965 patients. Fellow eyes routinely differ, so that result is not biologically plausible. Parsed per eye from the diagnostic keyword columns, the discordance is 17.1% (444 of 2603 two-eye patients), and 44.7% of patients carry differing per-eye keyword strings. A substantial number of eyes in the pooled cohort therefore carried their fellow eye’s diagnosis.

Failure mode 4: the community metric is not the quantity it names: The ODIR-2019 reference scorer evaluates f1_score with average=“micro” on the flattened eight-column binary label matrix thresholded at 0.5. That reduces identically to element-wise accuracy over the matrix, and the matrix is 85.5% zeros. A model that predicts no positive label scores 0.855. A model that is perfect on normal, DR and other but blind to all five rare classes scores 0.970 while its macro-F1 is 0.375. Every figure reported on that scale sits above a floor of 0.855 that requires no model capability to reach.

Failure mode 5: a silent position-embedding drop in the foundation-model baseline: The RETFound masked-autoencoder checkpoint was pretrained at 224 px and carries a position embedding of shape (1197, 1024); a ViT-Large fine-tuned at 384 px requires (1577, 1024). The widely copied non-strict load guarded by shape == shape rejects pos_embed silently. No exception, no warning, and the network trains with randomly initialized position embeddings. The RETFound arm of the original analysis was affected.

Table 5 gives the four-probe gate before and after the cohort rebuild described in the next subsection. Eye-label discordance rose from 0% to 17.1%. Stored resolution went from non-uniform to uniform. The class signal recoverable from anatomy-free color and field-of-view statistics fell from +23.8 to +8.2 percentage points over chance, and source–label leakage became structurally impossible, the rebuilt cohort drawing on exactly one source. We disclose the residual +8.2 percentage points rather than claim it is zero. With a single source it cannot be provenance information, but it remains an unexplained residual.

### 3.6. Corrected Results on the Reconstructed Cohort

Near-duplicate sweep: Seven near-duplicate pairs were found among the 5911 eyes. Three straddled the train/test boundary. Two of those three were exact matches, identical on both hashes with a pixel correlation of 1.0000, between patients 2487 and 3185, the same patient filed under two identifiers in ODIR-5K, which patient-level splitting could not catch because the identifiers differed. Removing the three affected test images changed macro-AUC by −0.0001, macro-F1 by −0.0002, macro-F1 excluding hypertensive retinopathy by −0.0002 and accuracy by −0.0007. Every value reported in this paper is from the full 872-image test set; the leakage is real, it is disclosed, and it is too small to move any conclusion. The pair list is released.

The cohort was rebuilt from ODIR-5K alone: labels parsed per eye from the diagnostic keyword columns rather than inherited from the patient-level columns, and the patient as the unit of every split. RFMiD was withheld entirely for external validation. APTOS was dropped, being the sole carrier of the 1024 px DR images; keeping it would have reinstated the confound. Nine architectures were trained under one protocol. Every estimate below comes from the corrected pipeline, and none of it revises a number in the preceding subsections, which report the pooled cohort.

Held-out internal test set: The set contained 872 eyes from 495 patients, all from ODIR-5K and patient-disjoint from every training and validation image. Of the 872, 754 (86.5%) shared a patient with another test image, which is why the resampling unit for every internal interval was the patient and not the image. Each architecture was scored here exactly once, using the single run selected on the reporting half of its validation split, and no threshold, weight or hyper-parameter was touched afterwards. The ensemble is the unweighted arithmetic mean of the nine architectures’ predicted probability vectors. No combination weights were fitted, so it summarizes the nine runs rather than constituting a tenth model.

The ensemble reached a macro-AUC of 0.930 (95% CI 0.911–0.948). Given equal prominence alongside it: macro-F1 0.686 (0.637–0.733), macro-F1 excluding hypertensive retinopathy 0.773 (0.724–0.816), accuracy 0.803 (0.772–0.832). The nine individual architectures spanned a macro-AUC of 0.863–0.924 and a macro-F1 of 0.602–0.678 (Table 6). Weighted-F1 appears in that table for comparability with prior literature and supports no statement here. Its agreement with accuracy to three decimal places is a rounding coincidence, not a transcription error: weighted-F1 converges on accuracy when per-class precision and recall are close within the classes carrying most of the mass, and normal (n=462) and DR (n=234) represent 80% of this test set. Of 2000 bootstrap replicates, the one class producing a degenerate replicate was hypertensive retinopathy, in two of 2000.

A macro-AUC of 0.930 and a macro-F1 of 0.686 are not in conflict. The 0.24 between them is the most informative single quantity in Table 6. AUC asks whether an affected eye is ranked above an unaffected one; F1 asks whether the arg-max of the softmax lands on the right class. The ensemble put true hypertensive-retinopathy eyes at a mean percentile of 0.82 on the hypertensive-retinopathy score (AUC 0.825), then emitted that label for 10 of 872 eyes, two of them correctly, for an F1 of 0.160. The same asymmetry, less extreme, held for glaucoma (AUC 0.924, F1 0.571) and AMD (AUC 0.941, F1 0.694). Figure 2 gives the one-versus-rest ROC curves; Figure 3 plots both quantities against class size, where AUC is nearly flat across the range and F1 is not. All three headline metrics are printed throughout, and none is selected by outcome.

Per-class recall, F1 and confusion structure: Per-class recall for the ensemble at the arg-max rule was: pathological myopia 0.974 (n=39), cataract 0.905 (n=42), normal 0.820 (n=462), diabetic retinopathy 0.803 (n=234), glaucoma 0.698 (n=43), AMD 0.676 (n=37) and hypertensive retinopathy 0.133 (n=15). These recalls are the sensitivities of Table S4, and they are single-sample point estimates. No resampling interval is attached: as descriptors of one operating point, they support no comparison for which an interval would be interpretable. Per-class F1 for the ensemble, with 95% patient-level bootstrap intervals, was pathological myopia 0.905 (0.800–0.978), normal 0.833 (0.802–0.862), diabetic retinopathy 0.828 (0.782–0.869), cataract 0.809 (0.703–0.893), AMD 0.694 (0.523–0.822), glaucoma 0.571 (0.432–0.694) and hypertensive retinopathy 0.160 (0.000–0.357). Every class but normal and diabetic retinopathy had n<100 and is flagged as such in Table 7; those recalls and F1 values are imprecise, and no comparison is made between two such classes. Only the ensemble row carries intervals, because 63 class-by-architecture intervals would invite exactly the comparison the design cannot support. The hypertensive-retinopathy entry is exploratory throughout: n=15, its interval includes zero, it is never reported without its count, and no *p*-value is computed for it anywhere in this paper.

Two patterns dominate the confusion structure (Figure 4). The normal row is the largest absolute source of false positives: of 462 normal eyes, 31 are labeled glaucoma and 24 diabetic retinopathy, and that is what holds glaucoma’s positive predictive value to 0.484 despite a recall of 0.698. The largest single off-diagonal cell is DR → normal, at 41 of 234. The 15 hypertensive-retinopathy eyes distribute as six to diabetic retinopathy, six to normal, one to AMD and two correct. Pathological myopia is correct in 38 of 39 eyes, cataract in 38 of 42 and AMD in 25 of 37. All cells are exact counts and no *p*-value is attached to any of them.

External validation on RFMiD: RFMiD (721 images) was never trained on and covers four of the seven classes. Cataract and glaucoma are absent from its label mapping, so no external claim is made for them, and every macro average here is taken over the four classes actually present: normal (n=401), diabetic retinopathy (n=240), pathological myopia (n=46) and AMD (n=34). On that set the ensemble reached a macro-AUC of 0.978 (95% CI 0.970–0.985), macro-F1 of 0.677 (0.621–0.730) and accuracy of 0.716 (0.684–0.748) (Table 8). Those three intervals resampled *images*, not patients. RFMiD distributes no patient identifiers—its identifier field holds 721 distinct values for 721 images—so the external resampling unit can only be the image. That is a weaker guarantee than the patient-level resampling used throughout the internal analysis, and the external intervals should be read as correspondingly optimistic about their own width.

The external macro-AUC exceeds the internal one. That comparison is not interpretable until it is made class for class. Averaging the internal one-versus-rest AUCs of the same four classes gives 0.942 internally against 0.978 externally: ranking performance is preserved on a set the models never saw, but on a four-class average that omits the three internal classes with the lowest per-class AUC. The operating point moves too. Accuracy is 0.716 externally against 0.803 internally despite three fewer classes, external DR recall is 0.988 at a precision of 0.563, and pathological myopia recall falls to 0.413 at a precision of 0.950. No threshold was refitted on RFMiD and no external example was used for selection. Three explanations fit the pattern: the four-class restriction just quantified, a shift in class prior between the two sets, and thresholds calibrated on the internal prior and left unchanged. They are *candidates*, not findings. No experiment here was designed to attribute the internal–external difference, and we report it as observed rather than as explained.

The external ordering inverts, and the inversion is a decision-threshold effect rather than better generalization: On the external set, RETFound has the *lowest* macro-AUC of the nine (0.911) and at the same time the *highest* macro-F1 (0.763) and accuracy (0.853). The metrics disagree because they measure different things on the same probability vectors: macro-AUC scores the ranking and is threshold-free, while macro-F1 and accuracy score where the arg-max lands. The external set contains 401 normal, 240 diabetic-retinopathy, 46 pathological-myopia and 34 AMD images. Eight of the nine architectures emit a diabetic-retinopathy label between 390 and 471 times, 1.6 to 2.0 times the true frequency of the class, while predicting normal 210 to 289 times against a true 401. RETFound emits 304 diabetic-retinopathy and 373 normal labels. Its arg-max therefore sits closer to the external class prior than any other architecture’s, so its decisions are better matched to this distribution while its ranking is the weakest of the nine. We report an operating-point effect and draw no conclusion from it about representation quality: the threshold-free comparison, the one that speaks to discrimination, places RETFound last both internally and externally. This is the divergence documented per class in Figure 3, appearing here between architectures, and a concrete reason to report macro-AUC and macro-F1 together rather than choose between them.

Between-architecture comparison: equivalence, not ranking: One deviation from the analysis plan is disclosed. DeLong’s test was pre-specified for macro-AUC contrasts; its variance estimator assumes independent observations, and 86.5% of the test images shared a patient with another. Every macro-AUC and macro-F1 contrast was therefore tested with the paired patient-level bootstrap, on the same shared resampling index matrix as every other interval reported here. Accuracy contrasts used McNemar’s test as pre-specified, and no contrast fell below the 30-discordant-pair floor. Holm correction was applied within each metric family of 36 pairs. The deviation widened every interval and is therefore conservative with respect to the reported contrasts.

The primary contrast is null. EfficientNet-B3 versus ViT-Base/16 gave Δmacro-AUC =−0.0123 (95% CI −0.0369 to +0.0142; p=0.36, uncorrected as the sole primary test). The interval contains zero. On the primary endpoint, the convolutional and transformer backbones are statistically indistinguishable. Accuracy within the same contrast differed by +4.2 percentage points (+1.3 to +7.2; McNemar p=0.004 uncorrected, p=0.096 after Holm correction within the accuracy family, 155 discordant pairs). This difference is reported but is not advanced as a positive finding: the primary endpoint is null, the contrast does not survive its own family’s correction, and accuracy at this prevalence structure is dominated by the two largest classes.

No architecture was declared superior. Across the nine, macro-AUC spanned 0.8628 to 0.9238, a total range of 0.0610, and macro-F1 spans 0.602 to 0.678. Of the 36 pairwise macro-AUC contrasts, 14 had bootstrap intervals excluding zero before correction and two survived Holm correction; both survivors pitted one end of the range against the other. Not one of the eight adjacent-rank macro-AUC contrasts came close. The largest adjacent difference was 0.0230, the smallest uncorrected *p* was 0.17, and all eight had Holm-corrected p=1.00 (Table S3). What the nine runs establish is a band, not an ordering: on this cohort, one training protocol applied to nine standard backbones produced a macro-AUC between 0.86 and 0.92 and macro-F1 between 0.60 and 0.68, with the ensemble a little above the band on both. Neither table ordering nor figure placement implies a ranking the design cannot support. Domain-specific pretraining did not outperform ImageNet pretraining here, and RETFound had the lowest macro-AUC and macro-F1 of the nine. Its own uncertainty applies with full force though: its macro-AUC differed from only two of the eight other architectures after Holm correction, four of its contrasts contained zero, and on the external set, it had the highest accuracy and macro-F1 of the nine. This is not the loading bug. The RETFound arm was retrained with the position-embedding failure repaired, the embedding bicubically interpolated from the 14×14 to the 24×24 grid.

Post hoc descriptive analyses: The two analyses that follow are **post hoc and were not pre-specified**. No claim, *p*-value or model selection rests on either. They are here because calibration and per-class operating characteristics are standard expectations in clinical reporting, and they are labeled so they cannot be mistaken for confirmatory results. On the held-out internal test set, the ensemble’s expected calibration error was 0.094 over 15 equal-width bins, its multi-class Brier score 0.322 and its negative log-likelihood 0.630. Mean predicted confidence was 0.724 against an accuracy of 0.803: the ensemble was *under*-confident by 0.079. No resampling intervals are attached to these single-sample summaries, and no temperature scaling or other recalibration was applied (Figure S3).

Table S4 gives sensitivity—per-class recall—with specificity, positive predictive value (PPV) and negative predictive value (NPV) for the ensemble at the arg-max rule, the same operating point the F1 values of Table 7 already describe. Predictive values are conditional on this cohort’s prevalence, which is set by dataset composition and not by any screening population. They do not transfer. Glaucoma reaches a sensitivity of 0.698 at a specificity of 0.961 yet a PPV of only 0.484: at this prevalence, the 31 false positives it draws from 462 normal eyes dominate everything else it confuses. Hypertensive retinopathy has a specificity of 0.991 and sensitivity of 0.133. The model almost never emits the label, which is what its F1 of 0.160 at n=15 reports, and no *p*-value is computed for it. Neither entry constitutes a screening claim, and no result reported here supports deployment.

Qualitative attribution maps: Figure 5 shows Grad-CAM overlays from a model trained on the rebuilt cohort. They are illustrative and carry no inferential weight. They are specifically not evidence that shortcut learning has been removed, as the caption sets out at length, because the confounded model of the original analysis also produced anatomically plausible maps.

### 3.7. Original Versus Corrected Estimates

Table 9 places the two analyses side by side. The middle column holds exactly the quantities reported in the preceding subsections of this Results section; the right-hand column those of the reconstructed cohort. These are not two attempts at the same measurement. They are measurements of different things on different cohorts, and the table is arranged so a reader can see which design choice moved which number.

The line of that table that bears most directly on the comparison is not a performance number. With Cramér’s V=0.9996 between acquisition source and class label, the pooled cohort supplies a variable that determines the label almost perfectly and is visible in the file header, in the stored resolution and in the corpus-level color statistics. A weighted-F1 of 0.9507 obtained on such a cohort does not measure how well a network reads retinal pathology. It very largely measures how well a network reads provenance. The DR recall of exactly 1.0000, identical across five architectures with quite different inductive biases, is the clearest single indication: networks that disagreed about everything else agreed perfectly about DR, because DR was the corpus and not the disease.

The movement from a weighted-F1 of 0.9507 to a macro-F1 of 0.686 is therefore *not* a loss of performance. These are different metrics computed on different cohorts under different labeling and splitting rules, and only the second is a valid estimate of anything. Weighted-F1 is dominated by the two largest classes and, on a cohort that was 62.5% DR, assigned a high score to the reproduction of a resolution cue. Macro-F1 weights all seven classes equally on a cohort in which the label is per eye and the source constant, so it is held down by the rare classes that the earlier figure absorbed into the average: glaucoma, AMD and, most of all, hypertensive retinopathy at n=15. The corrected estimates are lower, based on a smaller cohort and accompanied by wider intervals, and they are the estimates this study reports as valid. Table 9 is arranged so that both sets of estimates, and the design choices that separate them, can be read together.

Two further consequences follow. The pooled-cohort conclusion that a compact convolutional network outperforms a retinal foundation model and Vision Transformers does not survive the correction: on the rebuilt cohort, the primary contrast between EfficientNet-B3 and ViT-Base/16 has a macro-AUC difference of −0.0123 with an interval spanning zero, all eight adjacent-rank contrasts have Holm-corrected p=1.00, and the only contrasts that survive correction lie between the two extremes of a 0.061-wide band. The corrected finding is equivalence across nine backbones, not a ranking. And the RETFound arm of the original analysis trained with randomly initialized position embeddings, so its place at the bottom of that ordering was partly an artefact of a silent loading failure. Retrained with that failure repaired, it still sits at the low end internally while attaining the highest accuracy and macro-F1 of the nine on the external set. Neither result supports any claim of clinical utility or readiness for deployment, and no such claim is made.

## 4. Discussion

### 4.1. Principal Findings

Pooled-cohort analysis: The findings restated in this subsection are those of the pooled cohort, whose provenance confound is documented in Section 3.5. They are reinterpreted later in this Discussion, and the claim that any one architecture outperforms the others does not stand.

In this multi-source, five-fold cross-validation study, a compact convolutional network (EfficientNet-B3) achieved the strongest in-distribution performance for seven-class retinal disease classification, significantly exceeding a substantially larger retinal foundation model (RETFound ViT-Large; t=9.70; p<0.001), a Vision Transformer (ViT-Base/16; t=9.20; p<0.001), and Inception-V3 (t=8.38; p<0.001). The advantage was consistent across all five independent folds, with no fold reversing the ordering. Our findings demonstrate that larger retinal foundation models are not inherently superior to compact CNNs for heterogeneous retinal disease screening. For clinical practice, the salient implication is not which architecture “wins” in isolation, but that accurate simultaneous multi-disease screening can be delivered by lightweight models that are far easier to deploy at scale.

### 4.2. Reinterpretation of the Original Findings

The headline of the pooled-cohort analysis, accuracy of 95.53% and weighted-F1 of 0.9507 on the pooled 11,223-image cohort, measured provenance rather than pathology. Every diabetic-retinopathy image in that cohort came from a 1024 px corpus and every image of the six other classes from 512 px corpora, so the decision rule *width equals 1024 implies diabetic retinopathy* reproduces the label at 99.98% accuracy while reading zero retinal pixels. Source and label were near-deterministically dependent (Cramér’s V=0.9996), and diabetic-retinopathy recall was exactly 1.0000 for all five architectures. That value indicates a shortcut rather than discriminative performance. The reported figure estimates how well the networks recovered acquisition metadata. It is not a measure of retinal disease classification.

The architecture ranking the pooled-cohort analysis reported does not survive reconstruction. Nine architectures trained under one protocol on the reconstructed single-source cohort span 0.061 in macro-AUC end to end, and the pre-specified contrast is null: Δmacro-AUC =−0.0123 (95% CI [−0.0369,+0.0142]; p=0.36). Of the 36 pairwise contrasts, only two survive Holm correction, both of those at the extremes of the ranking, and all eight adjacent-rank contrasts have Holm-adjusted p=1.00. The claim that any architecture is superior to any other for this task is therefore not supported. The reconstructed analysis supports something weaker: under a common training protocol on this cohort, the nine architectures are statistically indistinguishable except at the extremes of a narrow performance range.

Set the confound aside and the original statistical analysis is still inadequate. Paired *t*-tests were computed across five cross-validation folds with df=4, treating the folds as independent samples when by construction they share training data and so violate the independence the test requires. The variance-corrected resampled *t*-test of Nadeau and Bengio applied to the same fold scores gave a variance multiplier of 0.455 and confidence intervals 1.51× wider than those reported. That correction alone removes significance from both contrasts that carried the pooled-cohort claim: ΔF1 =0.0075 against ViT-Base/16 and ΔF1 =0.0103 against Inception-V3. No multiplicity correction was applied to the four reported comparisons either, so the nominal *p*-values were optimistic on that count too. These are errors of inference rather than of arithmetic, and they would have inflated the original claim on an unconfounded cohort as well.

The RETFound result needs a correction of its own. A silent position-embedding load failure disadvantaged RETFound in the original analysis: the positional embeddings of the pretrained checkpoint never reached the fine-tuning model, the loading routine raised no error, and the comparison was therefore made against a partially initialized network. Retrained with that defect repaired, RETFound still ranked lowest of the nine architectures on the reconstructed cohort, at a macro-AUC of 0.863 but differed from only two of the eight others after Holm correction. The evidence supports a narrow claim: on this cohort, at this sample size, with this fine-tuning budget, retina-specific pretraining did not outperform ImageNet pretraining. It is not evidence that RETFound is a weak model. The original framing of the comparison is withdrawn.

### 4.3. Why the Corrected Numbers Are Lower, and Why That Is the Result

The corrected macro-F1 of 0.686 and the originally reported weighted-F1 of 0.9507 are not comparable quantities on comparable data. The averaging schemes differ: one weights every class equally, the other weights every class by its prevalence in a cohort in which diabetic retinopathy accounted for 62.5% of images. The cohorts differ too, in source composition, label granularity and unit of analysis. The apparent drop removes an artefact; it does not lose model performance, and the two figures are not comparable on a common axis. The pre-specified secondary macro-F1 excluding hypertensive retinopathy is 0.773 (95% CI [0.724,0.816]), reported in addition to the primary macro-F1 of 0.686 (95% CI [0.637,0.733]) and not as a substitute for it.

Discrimination on the reconstructed cohort is not low. The ensemble reaches a macro-AUC of 0.930 (95% CI [0.911,0.948]) on the held-out test set and 0.978 (95% CI [0.970,0.985]) on the external RFMiD evaluation. Both sit inside the range the published literature reports for multi-class fundus classification. The difference is that they were obtained on a cohort in which the label is not recoverable from acquisition metadata.

Arithmetic unrelated to model quality also bounds macro-F1 on this cohort from above. Hypertensive retinopathy contributes 15 eyes to the test set. A classifier perfect on the other six classes that missed every hypertensive-retinopathy eye would be bounded at a seven-class macro-F1 of 0.840 to 0.855: 0.840 if the 15 missed eyes distribute evenly across the six remaining classes, 0.855 if all are called normal. The spread is there because those misassigned eyes damage the precision of whichever class absorbs them, so the ceiling depends on where they land, and no single four-decimal value can be derived. Allowing the partial hypertensive-retinopathy performance actually observed (F1 =0.160) raises the bound to at most 0.877. Single-grader labeling lowers the attainable ceiling further by an amount we cannot quantify, since ODIR-5K provides no second read against which grader disagreement could be estimated.

The divergence between AUC and F1 across classes is a decision-threshold effect, not a failure of discrimination. Hypertensive retinopathy has an AUC of 0.825 and an F1 of 0.160 on 15 eyes; glaucoma an AUC of 0.924 and an F1 of 0.571; age-related macular degeneration an AUC of 0.941 and an F1 of 0.694. The model orders cases far better in each case than the single argmax operating point converts into correct hard assignments, and for the rarest class, a handful of false positives collapses precision. Report only threshold-dependent metrics and discrimination is understated; report only AUC and what the classifier delivers as a decision is overstated.

### 4.4. Clinical Implications

Pooled-cohort analysis: This subsection rests on the pooled cohort, whose provenance confound is documented in Section 3.5. The deployment framing below is not supported by the corrected analysis; see the reinterpretation earlier in this Discussion, and the Limitations.

For an ophthalmologist or a health system, the practical question is not which network achieves the highest benchmark score, but whether accurate multi-disease screening can be delivered where it is most needed. EfficientNet-B3 attains its performance with roughly 25-fold fewer parameters than the RETFound foundation model, translating into lower graphics-processing-unit (GPU) memory requirements and faster inference. Notably, in our classical-classifier experiments a support vector machine trained on EfficientNet-B3 features performed within 2.0 percentage points of the full network while running on a central processing unit alone, indicating that accurate triage need not depend on dedicated accelerator hardware.

Such resource efficiency has concrete deployment consequences. Community and primary-care screening clinics, rural hospitals, and low- and middle-income settings frequently lack the high-end GPUs, reliable connectivity, and technical staff that large foundation models presuppose. A compact model that runs on commodity workstations or embedded devices lowers both the capital cost of acquisition and the recurring cost of operation, widening access to automated triage precisely where ophthalmologist coverage is sparsest. In short, EfficientNet-B3 requires substantially fewer computational resources while achieving competitive-to-superior performance, making it attractive for deployment in regional screening programs and resource-limited healthcare systems, where it could extend specialist-level triage to currently underserved populations.

### 4.5. Domain-Specific Pretraining Does Not Guarantee Superior Performance

Several mechanistic explanations may account for RETFound’s unexpectedly lower performance. First, masked autoencoding pretraining emphasizes low-level texture and structural reconstruction, an objective that may not optimally prepare the model for the high-level categorical discriminations required in supervised seven-class classification. Second, RETFound’s very large capacity may be prone to suboptimal fine-tuning on our moderate-scale training set (approximately 35,300 images per fold)—particularly for the rare classes with fewer than 300 native images—despite layer-wise learning rate decay. In contrast, EfficientNet-B3’s compact, compound-scaled design is highly efficient for transfer learning on datasets of this size [37]. Third, RETFound’s self-supervised pretraining corpus was drawn largely from tertiary-referral clinical sources acquired under relatively standardized imaging conditions, which may have encoded acquisition-specific low-level statistics that do not transfer optimally to the heterogeneous acquisition environments of ODIR-5K, RFMiD, and APTOS 2019.

These findings are consistent with a growing body of evidence that foundation models do not universally outperform task-specific fine-tuned models, particularly when general-purpose pretrained networks are adequately trained on diverse, task-relevant data [48,49].

### 4.6. Diagnostic Overlap and Misclassification

The models’ errors concentrated at clinically meaningful boundaries rather than occurring at random. Hypertensive retinopathy and glaucoma share vascular and optic-disc features—arteriolar narrowing, arteriovenous nicking, peripapillary atrophy, and neuroretinal rim changes—that make their discrimination difficult even for experienced ophthalmologists on color fundus photography alone. Likewise, advanced hypertensive retinopathy and AMD can both produce posterior-pole pigmentary and exudative changes. That the misclassifications cluster at these ambiguous interfaces, and that Grad-CAM activations localized to the corresponding anatomical structures, suggests the networks are attending to genuinely diagnostic features rather than spurious correlations. It also implies a clinical ceiling: definitive discrimination of these entities may require ancillary data—intraocular pressure, optical coherence tomography, or blood-pressure history—that a fundus photograph cannot supply, and that a prudent screening system should flag such cases for clinician review rather than commit to a single label.

### 4.7. Comparison with Prior Work

Pooled-cohort analysis: The comparison below sets the pooled-cohort weighted-F1 of 0.9507 beside published benchmarks. That figure comes from the confounded cohort, so it is not a valid basis for comparison with any external result; the corrected estimates are given in the Original versus Corrected Estimates subsection of the Results.

Table S5 contextualizes our results against representative prior work. This table is intended for contextual positioning rather than direct rank comparison, given substantial heterogeneity in task definition, class count, dataset composition, and evaluation methodology. Our seven-class multi-source weighted-F1 of 0.9507 under rigorous five-fold cross-validation is competitive with or exceeds published benchmarks on the constituent datasets and represents a more challenging evaluation than most prior single-disease or single-dataset studies.

### 4.8. Limitations

Several limitations warrant emphasis, and we state them plainly. First, this was a retrospective analysis of publicly available benchmark datasets; performance on prospectively and consecutively acquired clinical cohorts is unknown and may be substantially lower. Second, no external, multicenter prospective validation was performed, the single most important step before any claim of clinical utility. Third, the public source datasets carry heterogeneous annotation protocols and diagnostic thresholds and are subject to referral and selection bias; they may not represent a general community screening population. Fourth, classification was image-only and unimodal: no optical coherence tomography, visual-field, intraocular-pressure, blood-pressure, or clinical-history data were incorporated, so the model cannot replicate the integrative diagnostic reasoning of a clinician. Fifth, we conducted no demographic subgroup analysis (age, sex, ethnicity) and no camera- or device-specific analysis; algorithmic fairness and robustness across patient subgroups and imaging hardware therefore remain uncharacterized and could conceal clinically important disparities. Sixth, the severe class imbalance meant that rare categories—most notably hypertensive retinopathy (28 test images)—were evaluated on small samples, and their per-class estimates should be regarded as preliminary. Seventh, the preprocessing and classical-machine-learning experiments used a single held-out split, and the Grad-CAM analysis was qualitative, without formal quantification against ophthalmologist-annotated lesion masks.

The reconstructed analysis adds the following limitations, each with the number that bounds it.

1. *No clinical claim is made.* There are no prospective data, no clinician comparator arm, no defined intended-use population. Class prevalence here is set by the composition of a public dataset, not by the epidemiology of any screening population, so no operating point reported here transfers to a screening or deployment setting and none of these results should be extrapolated to one.
2. *Glaucoma positive predictive value is 0.484.* On the held-out test set, 31 of 462 eyes labeled normal are assigned to glaucoma. In a screening population, where normal eyes vastly outnumber glaucomatous ones, that false-positive rate would generate more false alarms than true detections. That arithmetic is why no screening claim is made.
3. *Glaucoma from disc photographs is an unreliable reference standard.* Among glaucoma experts, interobserver agreement on whether a disc photograph shows glaucomatous damage is only moderate (median κ=0.50) [50], and among optometrists, general ophthalmologists and residents, the specificity of that judgment ranges from 47 to 60% [51]. Agreement on progressive disc change is lower still (κ=0.20 among glaucoma specialists) [52]. The label this study learns is itself noisy, so part of the residual error is irreducible against that reference.
4. *Hypertensive retinopathy without a blood-pressure measurement is not a diagnosis.* No systemic measurement accompanies these images, so the class as constituted here is a fundus appearance labeled by keyword rather than a clinical entity. On a purpose-built public hypertensive-retinopathy benchmark of 1000 images, the best-performing challenge entry reached an F1 of 0.612 [53]. Read the F1 of 0.160 obtained here on 15 eyes in a heavily imbalanced test set against that, not against the other six classes.
5. *Single-source training is a trade, not a strict improvement.* Restricting the cohort to one source makes source-label leakage structurally impossible: measured leakage is +0.00 percentage points and Cramér’s *V* between source and label is 0.0000 on the test set. It also means the study measures one acquisition protocol applied to one population. This design does not establish generalization across cameras, sites and populations, and none is claimed.
6. *A residual color-to-class dependence of +8.2 percentage points above chance remains*, down from +23.8 percentage points in the original cohort. Some of it is genuine clinical signal; several of these diseases do alter fundus coloration. An earlier draft attributed the residual to cataract-related yellowing, and direct measurement refuted that: cataract recall from color features alone is 0.12, below the 0.143 chance rate for seven classes. The source of the residual dependence is unidentified. We report it as an open finding rather than explain it away.
7. *The label mapping is fully auditable but was not clinically reviewed.* It comprises 155 distinct keyword strings, none mapping to more than one class, and can be verified in full by reading 155 rows without opening a single image. No ophthalmologist reviewed it and no image was re-graded for this study. Auditability is not clinical validation.
8. *The taxonomy exclusions are not neutral.* The excluded eyes include 161 with drusen among their findings (138 with drusen as the sole finding), 290 with epiretinal membrane, 27 with tessellated fundus and 25 with chorioretinal atrophy. Drusen are the hallmark of early age-related macular degeneration, so the AMD class here is keyword-stated AMD rather than the full early-to-late spectrum and, more consequentially, the normal class is free of drusen-bearing eyes that a clinician would decline to call normal. Both the AMD task and the normal-versus-disease task are easier here than in clinic.
9. *The single-label filter selected the least distinctive part of the hypertensive-retinopathy class.* It removed 43% of hypertensive-retinopathy eyes (83 of 193), and 67 of those 83 went because they also carried a diabetic-retinopathy label. What survived was hypertensive retinopathy without concurrent diabetic retinopathy: the presentations with the fewest cotton-wool spots and hemorrhages. The filter did not draw a random sample of the class. It systematically retained the least distinctive members, and the low F1 for this class must be read with that selection in mind.
10. *Multi-label classification is the clinically correct framing and is not attempted here.* Eyes routinely carry more than one finding, and forcing a single label discards that structure. This study is a single-label seven-class task by construction; its metrics do not estimate performance on the multi-label problem a clinical system would have to solve.
11. *The Grad-CAM maps are qualitative and provide no evidence regarding shortcut learning.* They are reported for transparency. They are also post hoc, were never validated against ophthalmologist-annotated lesion masks, and cannot establish that shortcut learning is absent. The strongest evidence on the point is internal to this work: attention maps that appeared plausible coexisted with the confounded model, which was classifying on image provenance.
12. *External validation is partial.* RFMiD supports only four of the seven classes, leaving three classes with no external estimate at all. It also distributes no patient identifiers, so the bootstrap confidence intervals for the external evaluation resample images rather than patients and are correspondingly optimistic wherever a patient contributes more than one image.
13. *Three near-duplicate pairs cross the train/test boundary.* The perceptual sweep found seven near-duplicate pairs in the cohort, three of them spanning the split; two were the same patient under two ODIR-5K identifiers. Excluding the affected test images moved no metric by more than 0.0007, so the reported values stand, but the cohort is not duplicate-free and is not described as such.
14. *The audit gate itself has limits and is not exhaustive.* Gate P2 read only the first 400 of the 5911 images rather than the full cohort. Gate P3 was a cross-validated random forest fitted to a random subsample of 2200 of the 5911 eyes, reported as a point estimate with no confidence interval. The protocol screened for gross source-label dependence. Passing it did not establish that no shortcut was present.

### 4.9. Future Directions

Several extensions follow directly from these limitations. The essential next step is prospective, multicenter external validation on consecutively acquired screening images spanning diverse cameras, sites, and populations. Beyond this, integrating color fundus photography with optical coherence tomography and other modalities would enable multimodal learning that better mirrors clinical decision-making, and would directly address the diagnostic overlap identified here. Linking image models to structured electronic health record data—visual acuity, intraocular pressure, and systemic comorbidities such as hypertension and diabetes—could further sharpen discrimination of the confusable entities. Finally, moving from cross-sectional classification toward longitudinal modeling of disease progression and conversion risk would address the questions clinicians most often ask, positioning compact, deployable models as tools not only for detection but for ongoing disease management.

## 5. Conclusions

The pooled-cohort conclusion did not stand. The claim that a compact convolutional network outperformed a retinal foundation model and two transformer baselines rested on a cohort in which acquisition source and diagnostic label were near-deterministically dependent, and on paired tests that treated cross-validation folds as independent and applied no multiplicity correction. Neither the effect nor the inference survived correction.

On the reconstructed, de-confounded cohort, nine architectures trained under a single protocol were statistically indistinguishable except at the extremes of a macro-AUC range spanning 0.061. Ensemble discrimination was a macro-AUC of 0.930 (95% CI [0.911,0.948]) internally and 0.978 (95% CI [0.970,0.985]) on external validation, with a macro-F1 of 0.686 (95% CI [0.637,0.733]). No architecture was declared superior. No clinical utility was claimed.

What transfers from this work is not a model but the pretraining audit protocol that identified the failure, together with the evidence that the failure was invisible to every conventional check applied to the pooled cohort. Cross-validated accuracy, per-class recall, confusion matrices and attention maps gave no indication of the problem while the label was being read from image metadata. Public multi-source fundus collections should therefore be screened before any model is trained on them, for source-label dependence, for label granularity and for the semantics of the metric being optimized, and the result of that screening should be reported alongside performance rather than assumed. A benchmark number unaccompanied by such a screen cannot be interpreted, being indistinguishable from the pooled-cohort number reported above.

## Figures and Tables

**Figure 1:**
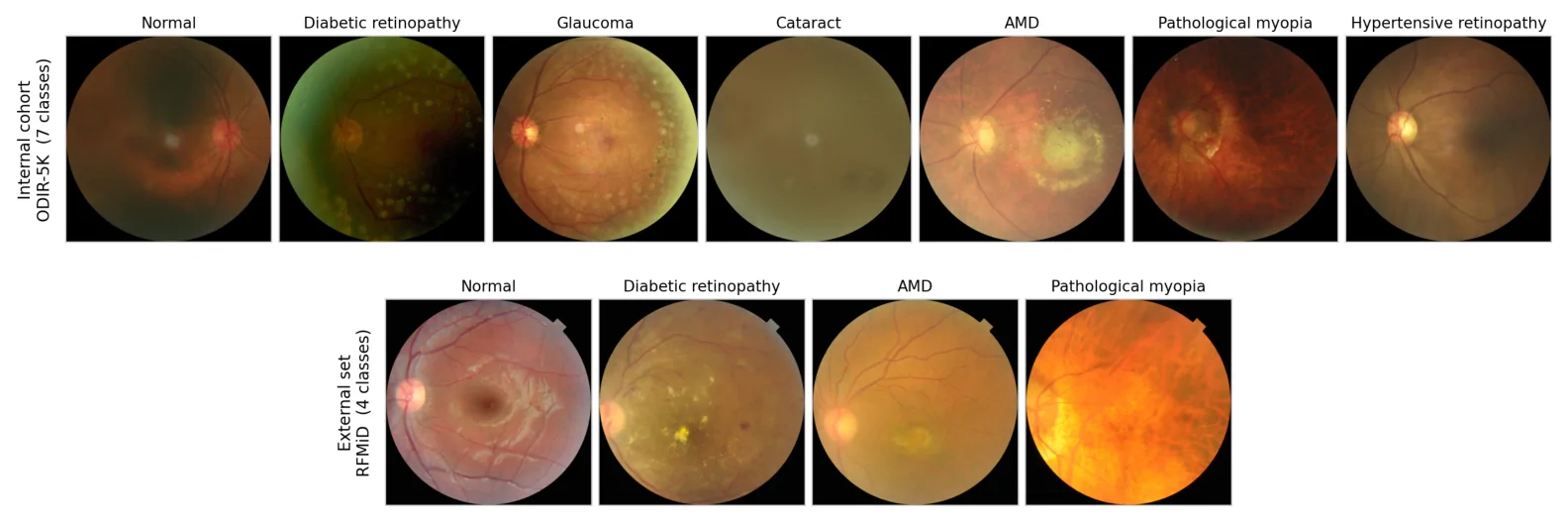
Representative fundus images after harmonization. Upper row: internal cohort (ODIR-5K), one image from each of the seven classes. Lower row: external set (RFMiD), the four classes its label set covers. Each image is the first of its class in manifest order and was not selected for appearance.

**Figure 2:**
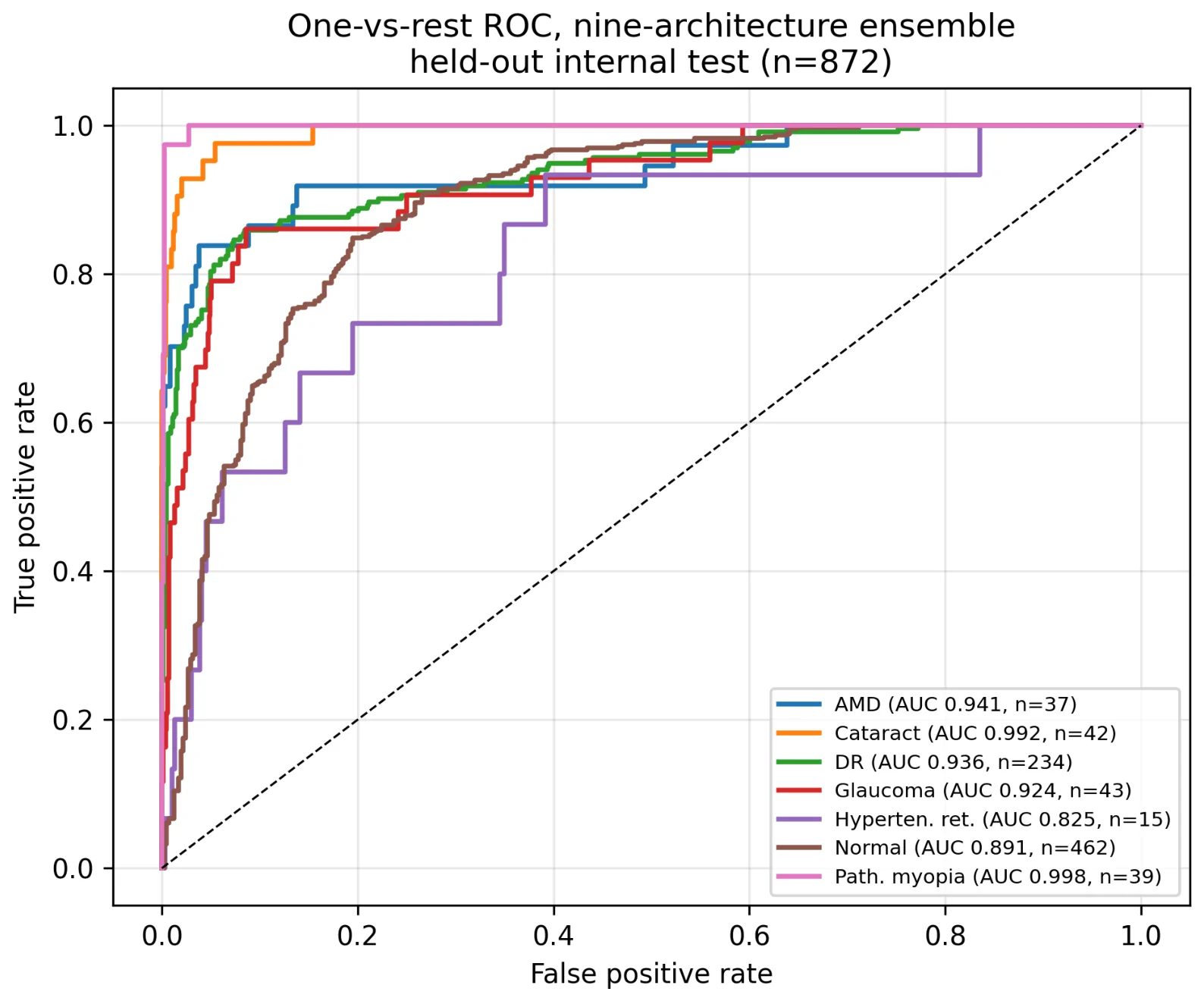
Corrected analysis: one-versus-rest ROC curves for the nine-architecture ensemble on the held-out internal test set (n=872 eyes from 495 patients). Class *n* is given in the legend. Ranking performance is high for every class, including hypertensive retinopathy (AUC 0.825) on 15 eyes, for which the arg-max F1 is 0.160. These curves and the per-class F1 values of Table 7 are two views of the same probability vectors. Curves for classes with n<100 are estimated from few positives and are correspondingly imprecise; they are shown for completeness, not as precise estimates. AMD = age-related macular degeneration; AUC = area under the receiver operating characteristic curve; DR = diabetic retinopathy.

**Figure 3:**
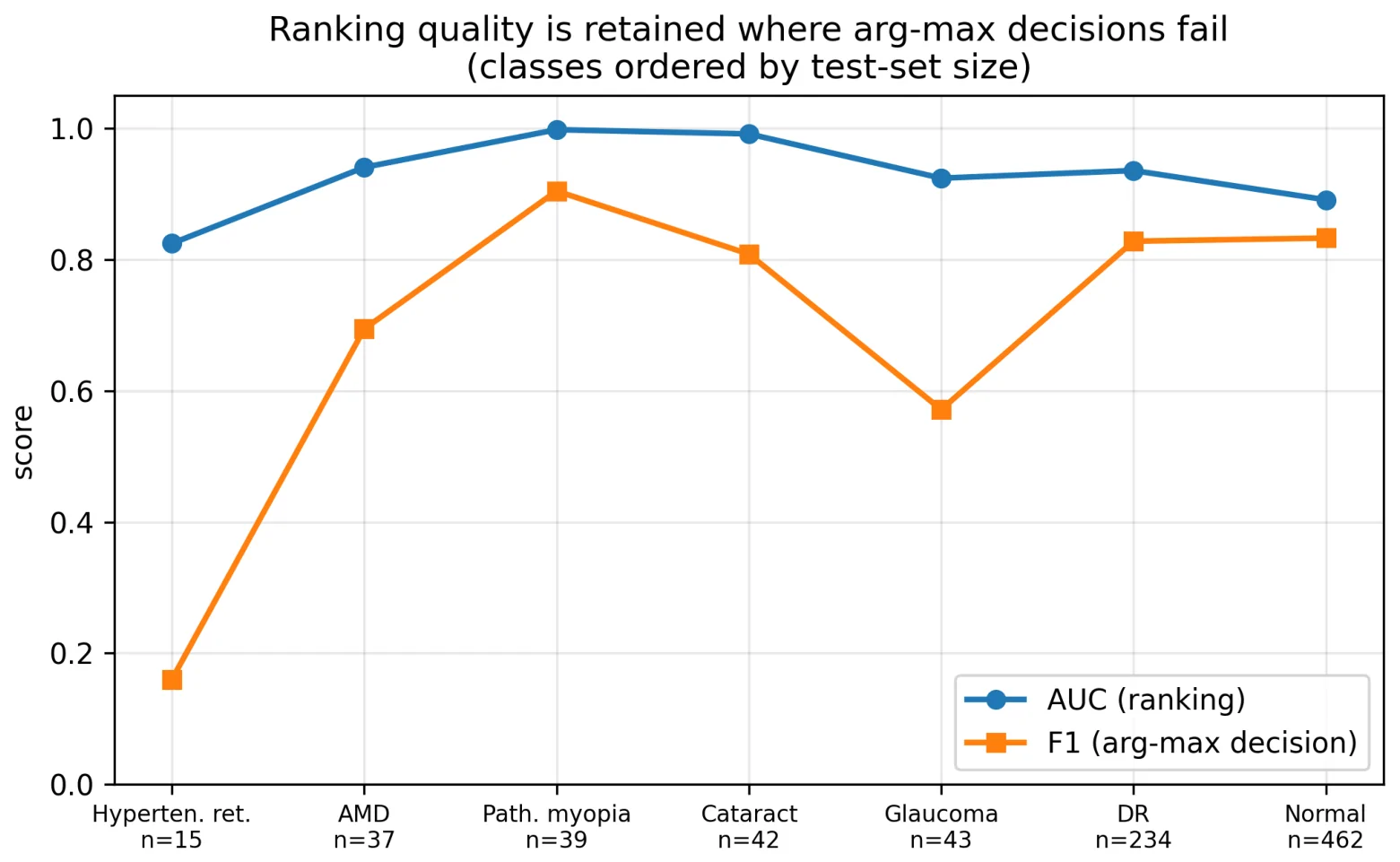
Corrected analysis: per-class one-versus-rest AUC and arg-max F1 for the ensemble on the held-out internal test set, with classes ordered by test-set size. AUC is approximately flat across the size range (0.825–0.998); F1 falls away as the class shrinks, reaching 0.160 for hypertensive retinopathy at n=15. Both curves are computed from the same probability vectors and differ only in whether a decision rule is applied. Pathological myopia shows that the pattern is driven by prevalence and separability jointly rather than by *n* alone: at n=39 it retains F1 0.905, because its appearance is distinctive enough to win the arg-max against 462 normal eyes. Point estimates are plotted; the corresponding 95% intervals for the ensemble are given in Table 7.

**Figure 4:**
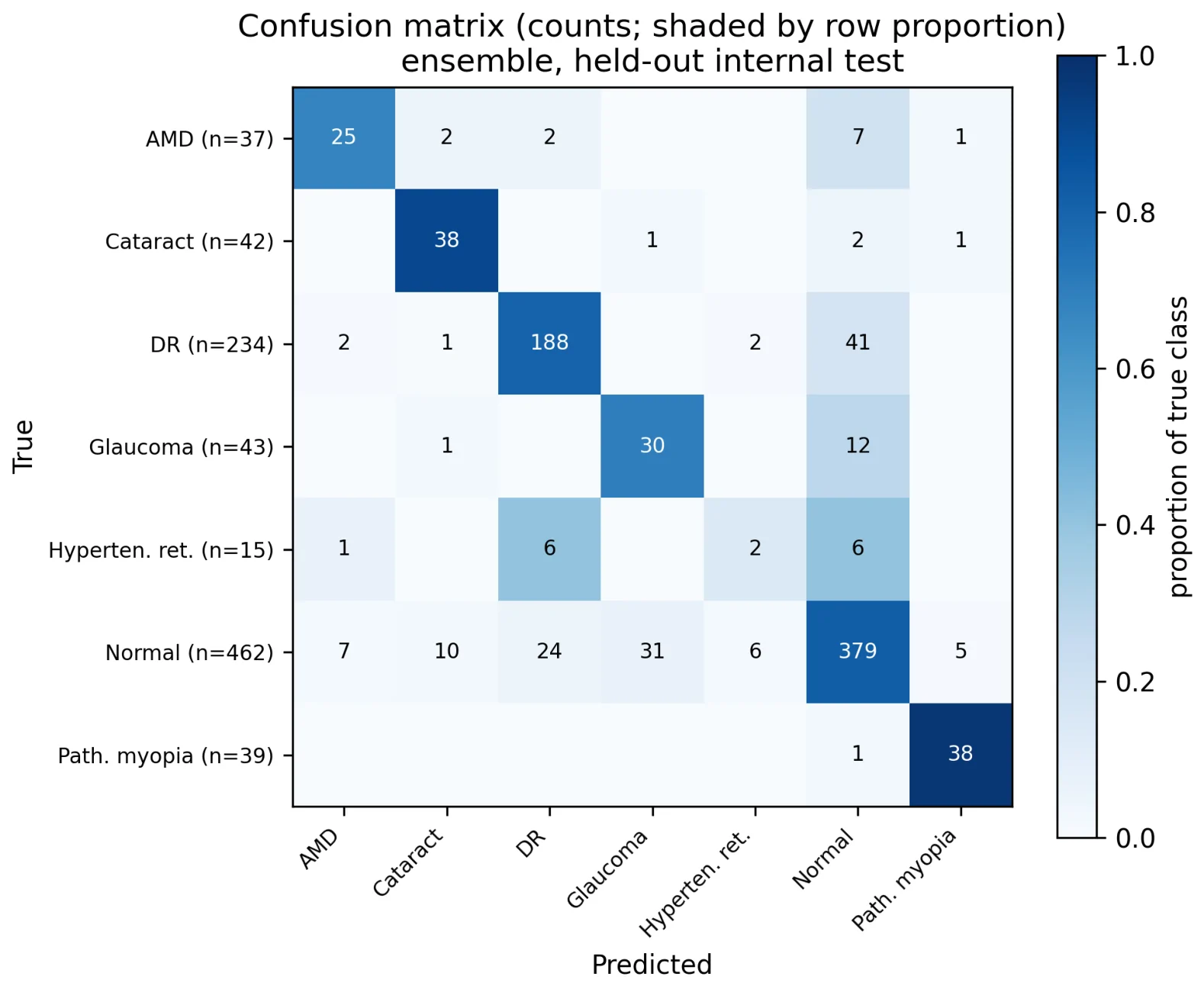
Corrected analysis: confusion matrix of the ensemble on the held-out internal test set (n=872 eyes). Cells give counts; shading is the row proportion, so color is comparable across rows of very different size. Two structures dominate. First, the normal row is the largest absolute source of false positives: 31 of 462 normal eyes are labeled glaucoma and 24 diabetic retinopathy, which is what holds glaucoma precision to 0.484 despite a recall of 0.698. Second, the 15 hypertensive-retinopathy eyes distribute as 6 to diabetic retinopathy, 6 to normal, 1 to AMD and 2 correct—the confusion the class definition predicts once eyes with concurrent diabetic retinopathy have been excluded. The matrix is descriptive and reports exact counts; no *p*-value is attached to any cell.

**Figure 5:**
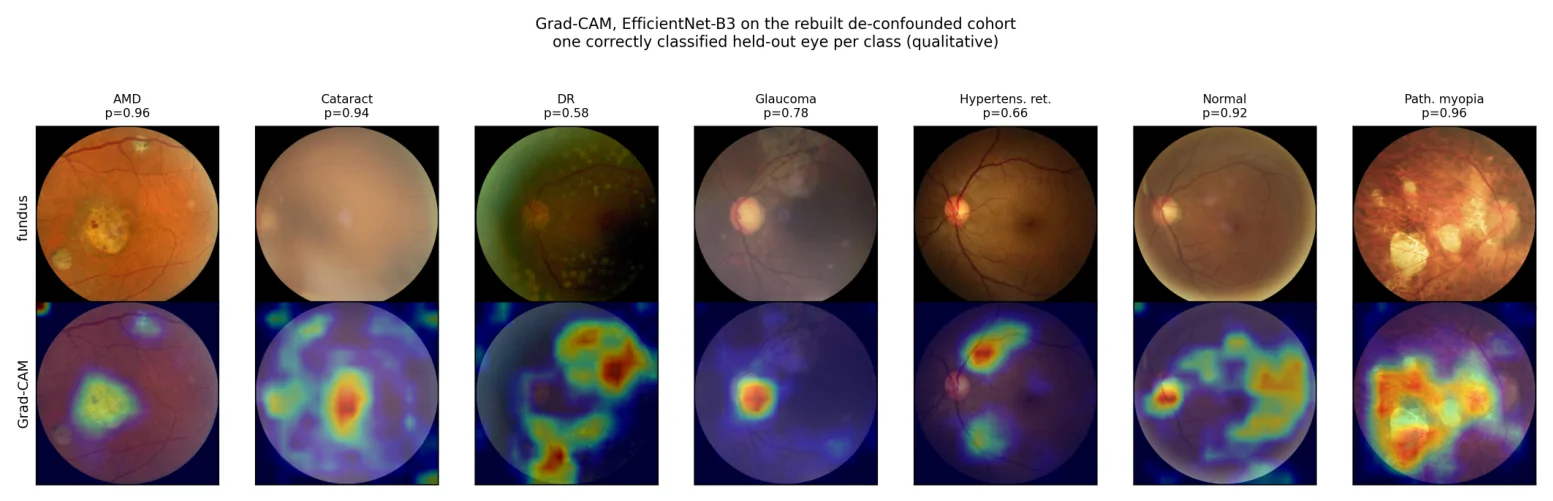
Corrected analysis: Grad-CAM overlays for a model trained on the rebuilt, de-confounded cohort: EfficientNet-B3, **fold-0** exponential moving-average weights at 512 px. These are one fold’s weights and *not* the nine-architecture ensemble; they are also a different run from the EfficientNet-B3 arm of Table 6, which reports the fold-2 run. The maps therefore illustrate a single model and characterize neither the ensemble nor the reported EfficientNet-B3 result. One correctly classified held-out eye is shown per class, selected as the first such eye in manifest order rather than chosen for appearance. The maps are qualitative and post hoc, and they have not been validated against lesion masks or any other reference annotation; no quantitative agreement statistic is computed for them. Grad-CAM and related attribution methods have documented validity problems, including failures of basic sanity checks such as model-parameter randomization. Attribution maps that appeared plausible coexisted with the confounded model of the original analysis, so a map that appears to localize on retinal structures is *not* evidence that shortcut learning is absent: an anatomically plausible map is fully compatible with a model exploiting a non-anatomical cue, and a map alone cannot distinguish the two. The evidence here that the shortcuts have been removed is structural (the single-source design) and measured (the four-probe gate of Table 5), not visual. This figure should not be cited as support for either claim.

**Table 1:** Class distribution of the pooled analytic cohort (11,223 unique patient-eye images) after deduplication and patient-level leakage control and of the fixed held-out test set (one image per patient). AMD = age-related macular degeneration. Bold marks the column totals. Percentages may not sum to 100.0 owing to rounding.

Disease Category	Total Images	% of Cohort	Held-Out Test
Diabetic retinopathy	7012	62.5	1051
Normal fundus	2874	25.6	431
Glaucoma	313	2.8	46
Cataract	301	2.7	45
AMD	278	2.5	41
Pathological myopia	253	2.3	37
Hypertensive retinopathy	192	1.7	28
**Total**	**11,223**	**100.0**	**1679**

**Table 2:** Provenance, annotation, de-identification and licensing of the public datasets used. APTOS 2019 contributed only to the pooled cohort and is not part of the reconstructed cohort.

Dataset	Access, Version and Date	Original Source and Annotation	De-Identification and License
ODIR-5K (ODIR-2019)	https://odir2019.grand-challenge.org/. Challenge release; the distributor issues no version number. Registration required. Accessed by the authors on 7 November 2025.	Collected by Shanggong Medical Technology Co., Ltd. (Shanghai, China) from multiple hospitals and medical centers across China. Includes 5000 patients, both eyes, with age and per-eye diagnostic keywords. Acquired on Canon, Zeiss and Kowa cameras, so image resolution varies within the dataset. Labeled by trained human readers under quality-control management into eight categories, using both eye images and patient age. One read per eye; no adjudication reported.	The distributor states that patient-identifying information has been removed and that release follows the ethical and privacy rules of the collecting jurisdiction. The official dataset page states no license or terms of use; access is granted on registration at the dataset’s official page and is governed by the terms accepted there. Research use permitted; redistribution not granted.
RFMiD	https://riadd.grand-challenge.org/. Version 1, published 3 February 2021 (Pachade et al., *Data* 2021;6(2):14, doi:10.3390/data6020014) [34]. A separate RFMiD 2.0 was released in 2023 and is *not* used here. Accessed by the authors on 20 April 2026.	Includes 3200 color fundus images from centers in India, acquired on three fundus camera models; 46 labeled conditions. Annotated by adjudicated consensus of two senior retinal experts. The present study uses the training partition and retains only images mapping to one of four of our seven classes.	Distributed de-identified by the publishing institutions. Creative Commons Attribution 4.0 International (CC BY 4.0): reuse and redistribution permitted with attribution.
APTOS 2019	https://www.kaggle.com/c/aptos2019-blindness-detection. Competition release, 2019; the host issues no version number. Accessed by the authors on 20 April 2026. Contributed to the pooled cohort only.	Organized by the Asia Pacific Tele-Ophthalmology Society; images contributed by Aravind Eye Hospital, India. Includes 3662 training images graded 0–4 for diabetic-retinopathy severity by clinical staff.	Distributed de-identified. No separate license is issued; use is governed by the competition rules accepted on the hosting platform. Redistribution not granted.

**Table 3:** Per-architecture settings for the reconstructed analysis. Every other hyper-parameter is shared, as described above. Effective batch is the micro-batch multiplied by the accumulation factor.

Architecture	Input (px)	Micro-Batch	Accumulation	Effective Batch
ConvNeXt-Small	512	16	2	32
ConvNeXt-Base	512	12	3	36
EfficientNet-B3	512	16	2	32
EfficientNetV2-S	512	16	2	32
DenseNet-121	512	24	1	24
Inception-V3	512	24	1	24
ViT-Base/16	384	16	2	32
Swin-Base	384	12	3	36
RETFound (ViT-L)	384	8	4	32

**Table 4:** Five-fold cross-validation classification performance. Mean and sample SD (denominator n−1=4) across five stratified folds. Bold indicates the highest value per metric column.

Model	Pretraining	Mean Acc	SD Acc	Mean F1	SD F1	Per-Fold F1 (Folds 0–4)
**EfficientNet-B3**	ImageNet-1K	**0.9553**	0.0026	**0.9507**	0.0029	[0.9469, 0.9482, 0.9527, 0.9529, 0.9527]
DenseNet-121	ImageNet-1K	0.9488	0.0058	0.9448	0.0058	[0.9400, 0.9482, 0.9372, 0.9500, 0.9485]
ViT-Base/16	ImageNet-21K/1K	0.9461	0.0036	0.9432	0.0031	[0.9390, 0.9421, 0.9424, 0.9455, 0.9470]
Inception-V3	ImageNet-1K	0.9468	0.0042	0.9404	0.0050	[0.9362, 0.9346, 0.9414, 0.9431, 0.9467]
RETFound ViT-L	Retinal CFP (1.6M)	0.9374	0.0059	0.9251	0.0076	[0.9122, 0.9277, 0.9250, 0.9288, 0.9319]

**Table 5:** The four-probe provenance gate, before and after the cohort rebuild. “Before” values are measured on the pooled benchmark of the pooled analysis and on ODIR-5K under the per-patient labelling scheme; “after” values are measured on the rebuilt single-source cohort. The probes are not of uniform strength and are not presented as though they were. P1 (per-eye label discordance) and P4 (source count) are exact enumerations over every eye in the cohort, so no resampling interval applies to them. P2 and P3 are not. In the after-rebuild run, P2 reads the stored width of only the first 400 of the 5911 images in manifest order, so “uniform (single width)” is a 400-image sample statement and not a census. P3 is a 300-tree random forest scored by 5-fold stratified cross-validation and fitted on a random subsample of 2200 of the 5911 eyes; its +8.2 percentage points is a point estimate reported without an interval, it varies with the subsample draw and the fold split, and that sampling uncertainty is not quantified anywhere in this paper. FOV = field of view; pp = percentage points.

Probe	Question/Pass Condition	Pooled Cohort	After Rebuild
**P1** labels	Is left/right label discordance biologically plausible? Pass if ≥5%.	0% across 1965 patients (patient labels stamped on both eyes)	17.1% (444/2603 two-eye patients)
**P2** resolution	Does exactly one stored width exist?	DR 1024 px vs. 512 px elsewhere; width==1024→DR is 99.98% accurate; Cramér’s V=0.9996; DR recall 1.0000 in 5/5 architectures	Uniform (single width)
**P3** color	Class recoverable from 22 anatomy-free color/FOV scalars? Pass if ≤ chance +0.15.	Class +23.8 pp over chance; source recovered at 96.95% balanced accuracy (chance 33.3%)	Class +8.2 pp over chance
**P4** source	Can source leak into the label at all? Pass if exactly one source.	Leakage present and measurable	Structurally impossible (single source)

**Table 6:** Corrected analysis: held-out internal test set (n=872 images from 495 patients, ODIR-5K, patient-disjoint from all training and validation data). Each architecture is scored *once*, by the single run selected on the reporting half of its validation split. Values are point estimates with 95% patient-level bootstrap intervals (B=2000, one shared resampling index matrix across all models and metrics). Rows are printed in the architecture order fixed before the test set was scored, not in performance order; the row ordering is not a claim, and no architecture is declared superior. Weighted-F1 is shown for comparability with prior literature and is excluded from every comparative statement. HR = hypertensive retinopathy. The bold marks the ensemble.

Architecture	Macro-AUC	Macro-F1	Macro-F1_eX-HR_	Weighted-F1	Accuracy
ConvNeXt-Small	0.919 [0.896, 0.940]	0.673 [0.624, 0.717]	0.745 [0.696, 0.787]	0.769 [0.739, 0.800]	0.757 [0.725, 0.788]
ConvNeXt-Base	0.924 [0.902, 0.944]	0.678 [0.629, 0.720]	0.756 [0.707, 0.797]	0.782 [0.750, 0.812]	0.775 [0.743, 0.806]
EfficientNet-B3	0.886 [0.859, 0.915]	0.653 [0.603, 0.698]	0.746 [0.697, 0.790]	0.769 [0.734, 0.802]	0.772 [0.738, 0.803]
EfficientNetV2-S	0.912 [0.894, 0.929]	0.634 [0.584, 0.679]	0.721 [0.667, 0.764]	0.737 [0.704, 0.771]	0.734 [0.701, 0.768]
DenseNet-121	0.889 [0.855, 0.920]	0.639 [0.589, 0.683]	0.722 [0.672, 0.765]	0.761 [0.729, 0.793]	0.759 [0.727, 0.791]
Inception-V3	0.912 [0.891, 0.932]	0.637 [0.583, 0.683]	0.709 [0.658, 0.750]	0.749 [0.717, 0.779]	0.745 [0.714, 0.777]
ViT-Base/16 (384)	0.898 [0.870, 0.924]	0.640 [0.596, 0.680]	0.738 [0.691, 0.780]	0.735 [0.702, 0.770]	0.729 [0.697, 0.764]
Swin-Base (384)	0.887 [0.860, 0.914]	0.606 [0.555, 0.647]	0.689 [0.635, 0.731]	0.709 [0.675, 0.743]	0.702 [0.668, 0.735]
RETFound (ViT-L, 384)	0.863 [0.828, 0.894]	0.602 [0.553, 0.645]	0.690 [0.636, 0.733]	0.680 [0.643, 0.715]	0.680 [0.644, 0.714]
**Ensemble of the nine**	**0.930 [0.911, 0.948]**	**0.686 [0.637, 0.733]**	**0.773 [0.724, 0.816]**	0.803 [0.771, 0.833]	**0.803 [0.772, 0.832]**

**Table 7:** Corrected analysis: per-class F1 on the held-out internal test set, with class *n*, and the ensemble’s per-class recall at the same arg-max operating point. Exploratory: counts and intervals only, no *p*-values. Rows and columns with n<100 are flagged; point estimates for those classes are imprecise and differences between architectures are not interpretable at these sample sizes. The last lines give the ensemble’s 95% patient-level bootstrap interval, its recall and its one-versus-rest AUC for the same class; recall is a single-sample point estimate carried without an interval. No *p*-value is computed for hypertensive retinopathy anywhere in this paper. AMD = age-related macular degeneration; DR = diabetic retinopathy; HR = hypertensive retinopathy. The bold marks the ensemble.

Architecture	Normal	DR	Cataract ^†^	Glaucoma ^†^	Path. Myopia ^†^	AMD ^†^	HR ^†^
	(n=462)	(n=234)	(n=42)	(n=43)	(n=39)	(n=37)	(n=15)
ConvNeXt-Small	0.783	0.815	0.718	0.528	0.905	0.722	0.240
ConvNeXt-Base	0.805	0.810	0.787	0.588	0.894	0.649	0.211
EfficientNet-B3	0.814	0.753	0.804	0.547	0.881	0.677	0.091
EfficientNetV2-S	0.765	0.748	0.760	0.510	0.874	0.667	0.114
DenseNet-121	0.794	0.790	0.750	0.505	0.826	0.667	0.138
Inception-V3	0.784	0.765	0.738	0.500	0.835	0.630	0.207
ViT-Base/16 (384)	0.766	0.722	0.831	0.509	0.905	0.692	0.054
Swin-Base (384)	0.737	0.725	0.706	0.479	0.854	0.634	0.105
RETFound (ViT-L, 384)	0.725	0.618	0.812	0.495	0.886	0.603	0.077
**Ensemble F1**	**0.833**	**0.828**	**0.809**	**0.571**	**0.905**	**0.694**	**0.160**
95% CI	[0.802, 0.862]	[0.782, 0.869]	[0.703, 0.893]	[0.432, 0.694]	[0.800, 0.978]	[0.523, 0.822]	[0.000, 0.357]
Recall (ensemble)	0.820	0.803	0.905	0.698	0.974	0.676	0.133
AUC (ensemble)	0.891	0.936	0.992	0.924	0.998	0.941	0.825

^†^ n<100; interval width exceeds ±0.10.

**Table 8:** Corrected analysis: external validation on RFMiD (n=721 images, never trained on; four of the seven classes present, and all macro averages are over those four). Point estimates; the ensemble’s 95% bootstrap intervals are given in the text, and per-architecture intervals are not reported because no comparison or selection was made on that set. RFMiD distributes no patient identifiers, so the resampling unit for the external intervals is the *image* and not the patient, which is a weaker guarantee than the patient-level resampling used internally. No threshold was refitted on RFMiD, and no architecture is declared superior on this set either. The bold marks the ensemble.

Architecture	Macro-AUC	Macro-F1	Weighted-F1	Accuracy
ConvNeXt-Small	0.955	0.667	0.690	0.687
ConvNeXt-Base	0.932	0.632	0.686	0.684
EfficientNet-B3	0.926	0.593	0.655	0.657
EfficientNetV2-S	0.961	0.654	0.677	0.677
DenseNet-121	0.932	0.646	0.729	0.724
Inception-V3	0.945	0.639	0.679	0.674
ViT-Base/16 (384)	0.932	0.670	0.741	0.737
Swin-Base (384)	0.939	0.627	0.639	0.638
RETFound (ViT-L, 384)	0.911	0.763	0.853	0.853
**Ensemble of the nine**	**0.978**	**0.677**	0.717	0.716

**Table 9:** The pooled-cohort analysis and the reconstructed analysis, side by side. The two columns are not comparable as estimates of a common quantity: they differ in cohort, in label unit, in split unit, in the number of architectures and in the reported metric. The middle column reproduces the figures reported earlier in this Results section without alteration. CI = confidence interval; DR = diabetic retinopathy.

Quantity	Pooled Cohort	After Reconstruction
Cohort	11,223 pooled patient-eye images	5911 ODIR-5K eyes
Sources	3 (ODIR-5K, RFMiD, APTOS)	1 (ODIR-5K); RFMiD reserved for external validation, APTOS not used
Label unit	Per-patient diagnosis columns applied to both eyes	Per eye, parsed from the diagnostic keyword columns
Split unit	Patient-eye	Patient
Source-to-label leakage	Present: Cramér’s V=0.9996 between source and label	Cramér’s V=0.0000 on the test set; structurally impossible with one source
Headline metric	Weighted-F1 0.9507 (accuracy 95.53%), 5-fold cross-validation	Macro-F1 0.686 (95% CI 0.637–0.733) with macro-AUC 0.930 (0.911–0.948), held-out test
DR recall	1.0000	0.803
Architectures	5	9
Held-out test set	Constructed (1679 images) but not reported; all reported values are cross-validation means	Reported; 872 images from 495 patients, each architecture scored once
Architecture ranking	EfficientNet-B3 declared superior	No architecture declared superior; nine backbones span 0.061 macro-AUC and the primary contrast is null

## Data Availability

All data analyzed in this study are publicly available, de-identified fundus image datasets: ODIR-5K (https://odir2019.grand-challenge.org/, accessed on 7 November 2025), RFMiD (https://riadd.grand-challenge.org/, accessed on 20 April 2026), and APTOS 2019 (https://www.kaggle.com/c/aptos2019-blindness-detection, accessed on 20 April 2026). The derived leakage-controlled fold assignments and the code used for preprocessing, training, and evaluation are available from the corresponding author on reasonable request. Full provenance, versioning, access, annotation, de-identification and license details for every dataset are given in Section 2.3. No images are redistributed: only ODIR-5K’s companion RFMiD carries a license permitting redistribution, and this study releases code, label-mapping rules, the manifest schema, split assignments and per-sample prediction arrays only.

## Supplementary Materials

The following supporting information can be downloaded at https://www.mdpi.com/article/10.3390/diagnostics16193109/s1: Table S1, Architecture summary; Table S2, Pairwise paired *t*-tests results; Table S3, Corrected analysis: pairwise comparisons on the held-out internal test set; Table S4, Corrected analysis: ensemble operating characteristics on the held-out internal test set at the arg-max decision rule; Table S5, Comparison with representative prior work on automated retinal disease classification; Figure S1, One-versus-rest AUC-ROC curves for all five architectures (fold 0 test set); Figure S2, Grad-CAM activation maps for EfficientNet-B3 (fold 3 checkpoint); Figure S3, Corrected analysis: reliability diagram for the nine-architecture ensemble on the held-out internal test set (*n* = 872 eyes), with predicted confidence binned into 15 equal-width bins.

## Abbreviations and Acronyms

AMD = age-related macular degeneration; AUC = area under the receiver operating characteristic curve; CNN = convolutional neural network; DR = diabetic retinopathy; Grad-CAM = gradient-weighted class activation mapping; MAE = masked autoencoder; SD = standard deviation; ViT = Vision Transformer.

## References

[1] World Health Organization (2019). World Report on Vision.

[2] Gulshan V., Peng L., Coram M., Stumpe M.C., Wu D., Narayanaswamy A., Venugopalan S., Widner K., Madams T., Cuadros J. (2016). Development and Validation of a Deep Learning Algorithm for Detection of Diabetic Retinopathy in Retinal Fundus Photographs. JAMA.

[3] Abramoff M.D., Lavin P.T., Birch M., Shah N., Folk J.C. (2018). Pivotal Trial of an Autonomous AI-Based Diagnostic System for Detection of Diabetic Retinopathy in Primary Care Offices. npj Digit. Med..

[4] Ting D.S.W., Cheung C.Y.L., Lim G., Tan G.S.W., Quang N.D., Gan A., Hamzah H., Garcia-Franco R., Yeo I.Y.S., Lee S.Y. (2017). Development and Validation of a Deep Learning System for Diabetic Retinopathy and Related Eye Diseases Using Retinal Images From Multiethnic Populations With Diabetes. JAMA.

[5] De Fauw J., Ledsam J.R., Romera-Paredes B., Nikolov S., Tomasev N., Blackwell S., Askham H., Glorot X., O’donoghue B., Visentin D. (2018). Clinically Applicable Deep Learning for Diagnosis and Referral in Retinal Disease. Nat. Med..

[6] Grassmann F., Mengelkamp J., Brandl C., Harsch S., Zimmermann M.E., Linkohr B., Peters A., Heid I.M., Palm C., Weber B.H. (2018). A Deep Learning Algorithm for Prediction of Age-Related Eye Disease Study Severity Scale for Age-Related Macular Degeneration From Color Fundus Photography. Ophthalmology.

[7] Li Z., He Y., Keel S., Meng W., Chang R.T., He M. (2018). Efficacy of a Deep Learning System for Detecting Glaucomatous Optic Neuropathy Based on Color Fundus Photographs. Ophthalmology.

[8] Ting D.S.W., Pasquale L.R., Peng L., Campbell J.P., Lee A.Y., Raman R., Tan G.S.W., Schmetterer L., A Keane P., Wong T.Y. (2019). Artificial intelligence and deep learning in ophthalmology. Br. J. Ophthalmol..

[9] Li T., Bo W., Hu C., Kang H., Liu H., Wang K., Fu H. (2021). Applications of deep learning in fundus images: A review. Med. Image Anal..

[10] Gargeya R., Leng T. (2017). Automated Identification of Diabetic Retinopathy Using Deep Learning. Ophthalmology.

[11] Burlina P.M., Joshi N., Pekala M., Pacheco K.D., Freund D.E., Bressler N.M. (2017). Automated Grading of Age-Related Macular Degeneration From Color Fundus Images Using Deep Convolutional Neural Networks. JAMA Ophthalmol..

[12] Peng Y., Dharssi S., Chen Q., Keenan T.D., Agrón E., Wong W.T., Chew E.Y., Lu Z. (2019). DeepSeeNet: A Deep Learning Model for Automated Classification of Patient-based Age-related Macular Degeneration Severity from Color Fundus Photographs. Ophthalmology.

[13] Cen L.P., Ji J., Lin J.W., Ju S.T., Lin H.J., Li T.P., Wang Y., Yang J.F., Liu Y.F., Tan S. (2021). Automatic detection of 39 fundus diseases and conditions in retinal photographs using deep neural networks. Nat. Commun..

[14] Poplin R., Varadarajan A.V., Blumer K., Liu Y., McConnell M.V., Corrado G.S., Peng L., Webster D.R. (2018). Prediction of cardiovascular risk factors from retinal fundus photographs via deep learning. Nat. Biomed. Eng..

[15] Wong T.Y., Mitchell P. (2004). Hypertensive Retinopathy. N. Engl. J. Med..

[16] Krause J., Gulshan V., Rahimy E., Karth P., Widner K., Corrado G.S., Peng L., Webster D.R. (2018). Grader Variability and the Importance of Reference Standards for Evaluating Machine Learning Models for Diabetic Retinopathy. Ophthalmology.

[17] Dosovitskiy A., Beyer L., Kolesnikov A., Weissenborn D., Zhai X., Unterthiner T., Dehghani M., Minderer M., Heigold G., Gelly S. An Image Is Worth 16 × 16 Words: Transformers for Image Recognition at Scale. Proceedings of the International Conference on Learning Representations (ICLR).

[18] Bommasani R., Hudson D.A., Adeli E., Altman R., Arora S., von Arx S., Bernstein M.S., Bohg J., Bosselut A., Brunskill E. (2021). On the Opportunities and Risks of Foundation Models. arXiv.

[19] Zhou Y., Chia M.A., Wagner S.K., Ayhan M.S., Williamson D.J., Struyven R.R., Liu T., Xu M., Lozano M.G., Woodward-Court P. (2023). A Foundation Model for Generalizable Disease Detection From Retinal Images. Nature.

[20] Liu X., Faes L., Kale A.U., Wagner S.K., Fu D.J., Bruynseels A., Mahendiran T., Moraes G., Shamdas M., Kern C. (2019). A comparison of deep learning performance against health-care professionals in detecting diseases from medical imaging: A systematic review and meta-analysis. Lancet Digit. Health.

[21] Nagendran M., Chen Y., Lovejoy C.A., Gordon A.C., Komorowski M., Harvey H., Topol E.J., A Ioannidis J.P., Collins G.S., Maruthappu M. (2020). Artificial intelligence versus clinicians: Systematic review of design, reporting standards, and claims of deep learning studies. BMJ.

[22] Geirhos R., Jacobsen J.H., Michaelis C., Zemel R., Brendel W., Bethge M., Wichmann F.A. (2020). Shortcut learning in deep neural networks. Nat. Mach. Intell..

[23] Zech J.R., Badgeley M.A., Liu M., Costa A.B., Titano J.J., Oermann E.K. (2018). Variable generalization performance of a deep learning model to detect pneumonia in chest radiographs: A cross-sectional study. PLoS Med..

[24] DeGrave A.J., Janizek J.D., Lee S.I. (2021). AI for radiographic COVID-19 detection selects shortcuts over signal. Nat. Mach. Intell..

[25] Badgeley M.A., Zech J.R., Oakden-Rayner L., Glicksberg B.S., Liu M., Gale W., McConnell M.V., Percha B., Snyder T.M., Dudley J.T. (2019). Deep learning predicts hip fracture using confounding patient and healthcare variables. npj Digit. Med..

[26] Kapoor S., Narayanan A. (2023). Leakage and the reproducibility crisis in machine-learning-based science. Patterns.

[27] Yagis E., Atnafu S.W., García Seco de Herrera A., Marzi C., Scheda R., Giannelli M., Tessa C., Citi L., Diciotti S. (2021). Effect of data leakage in brain MRI classification using 2D convolutional neural networks. Sci. Rep..

[28] Tampu I.E., Eklund A., Haj-Hosseini N. (2022). Inflation of test accuracy due to data leakage in deep learning-based classification of OCT images. Sci. Data.

[29] Varoquaux G., Cheplygina V. (2022). Machine learning for medical imaging: Methodological failures and recommendations for the future. npj Digit. Med..

[30] Roberts M., Driggs D., Thorpe M., Gilbey J., Yeung M., Ursprung S., Aviles-Rivero A.I., Etmann C., McCague C., Beer L. (2021). Common pitfalls and recommendations for using machine learning to detect and prognosticate for COVID-19 using chest radiographs and CT scans. Nat. Mach. Intell..

[31] Mongan J., Moy L., Kahn C.E.J. (2020). Checklist for Artificial Intelligence in Medical Imaging (CLAIM): A Guide for Authors and Reviewers. Radiol. Artif. Intell..

[32] Collins G.S., Moons K.G.M., Dhiman P., Riley R.D., Beam A.L., Van Calster B., Ghassemi M., Liu X., Reitsma J.B., van Smeden M. (2024). TRIPOD+AI statement: Updated guidance for reporting clinical prediction models that use regression or machine learning methods. BMJ.

[33] (2019). International Competition on Ocular Disease Intelligent Recognition. https://odir2019.grand-challenge.org/.

[34] Pachade S., Porwal P., Thulkar D., Kokare M., Deshmukh G., Sahasrabuddhe V., Giancardo L., Quellec G., Mériaudeau F. (2021). Retinal Fundus Multi-Disease Image Dataset (RFMiD): A Dataset for Multi-Disease Detection Research. Data.

[35] Kaggle (2019). APTOS 2019 Blindness Detection. https://www.kaggle.com/c/aptos2019-blindness-detection.

[36] Graham B. (2015). Kaggle Diabetic Retinopathy Detection Competition Report.

[37] Tan M., Le Q.V. EfficientNet: Rethinking Model Scaling for Convolutional Neural Networks. Proceedings of the International Conference on Machine Learning (ICML).

[38] Huang G., Liu Z., van der Maaten L., Weinberger K.Q. (2017). Densely Connected Convolutional Networks. Proceedings of the IEEE Conference on Computer Vision and Pattern Recognition (CVPR).

[39] Szegedy C., Vanhoucke V., Ioffe S., Shlens J., Wojna Z. (2016). Rethinking the Inception Architecture for Computer Vision. Proceedings of the IEEE Conference on Computer Vision and Pattern Recognition (CVPR).

[40] Dietterich T.G. (1998). Approximate Statistical Tests for Comparing Supervised Classification Learning Algorithms. Neural Comput..

[41] Holm S. (1979). A simple sequentially rejective multiple test procedure. Scand. J. Stat..

[42] Selvaraju R.R., Cogswell M., Das A., Vedantam R., Parikh D., Batra D. (2017). Grad-CAM: Visual Explanations From Deep Networks via Gradient-Based Localization. Proceedings of the IEEE International Conference on Computer Vision (ICCV).

[43] Nadeau C., Bengio Y. (2003). Inference for the generalization error. Mach. Learn..

[44] McNemar Q. (1947). Note on the sampling error of the difference between correlated proportions or percentages. Psychometrika.

[45] Efron B. (1987). Better Bootstrap Confidence Intervals. J. Am. Stat. Assoc..

[46] DeLong E.R., DeLong D.M., Clarke-Pearson D.L. (1988). Comparing the areas under two or more correlated receiver operating characteristic curves: A nonparametric approach. Biometrics.

[47] Clopper C.J., Pearson E.S. (1934). The use of confidence or fiducial limits illustrated in the case of the binomial. Biometrika.

[48] Raghu M., Zhang C., Kleinberg J., Bengio S. Transfusion: Understanding Transfer Learning for Medical Imaging. Proceedings of the Advances in Neural Information Processing Systems (NeurIPS).

[49] Ke A., Ellsworth W., Banerjee O., Ng A.Y., Rajpurkar P. CheXtransfer: Performance and Parameter Efficiency of ImageNet Models for Chest X-ray Interpretation. Proceedings of the Conference on Health, Inference, and Learning (CHIL).

[50] Varma R., Steinmann W.C., Scott I.U. (1992). Expert Agreement in Evaluating the Optic Disc for Glaucoma. Ophthalmology.

[51] Abrams L.S., Scott I.U., Spaeth G.L., Quigley H.A., Varma R. (1994). Agreement among Optometrists, Ophthalmologists, and Residents in Evaluating the Optic Disc for Glaucoma. Ophthalmology.

[52] Jampel H.D., Friedman D., Quigley H., Vitale S., Miller R., Knezevich F., Ding Y. (2009). Agreement Among Glaucoma Specialists in Assessing Progressive Disc Changes From Photographs in Open-Angle Glaucoma Patients. Am. J. Ophthalmol..

[53] Qian B., Wang X., Guan Z., Yang D., Ran A., Li T., Wang Z., Wen Y., Shu X., Xie J. (2025). HRDC challenge: A public benchmark for hypertension and hypertensive retinopathy classification from fundus images. Vis. Comput..

