# Supplementary material for: Comparative Evaluation of EfficientNet-B3, Vision Transformers, and RETFound for Seven-Category Retinopathy Fundus Image Classification
Source: Diagnostics (Basel). 2026 Sep 24;16(19):3109. doi: 10.3390/diagnostics16193109 (PMC13664116; doi:10.3390/diagnostics16193109)
Supplement: Supplementary file 1 [file diagnostics-16-03109-s001.zip › diagnostics-4519626-supplementary.pdf]

Supplementary Materials

Supplementary material for *Comparative Evaluation of EfficientNet-B3, Vision Transforers, and RETFound for Seven-Category Retinopathy Fundus Image Classification*. Tables S1–S2 and Figures S1–S2 belong to the pooled-cohort analysis; that cohort's provenance confound is documented in the main text. Tables S3–S5 and Figure S3 report detail of the corrected analysis.

Table S1. Architecture summary. CNN = convolutional neural network; ViT = Vision Transformer; CFP = color fundus photography; MAE = masked autoencoder.

Model	Type	Parameters	Pretraining data	Key mechanism
EfficientNet-B3	CNN	12M	ImageNet-1K	Compound scaling
DenseNet-121	CNN	8M	ImageNet-1K	Dense skip connections
Inception-V3	CNN	23M	ImageNet-1K	Multi-scale convolutions
ViT-Base/16	ViT	86M	ImageNet-21K/1K	Patch self-attention
RETFound ViT-L	ViT	307M	Retinal CFP (1.6M)	MAE pretraining

Table S2. Pairwise paired *t*-test results: EfficientNet-B3 vs. each comparator (d*f*=4, two-tailed, sample SD). ΔF1 = EfficientNet-B3 mean fold F1 minus comparator mean fold F1. The 95% CI is computed from the paired-difference SE with $t_{0.9754}$=2.776; Cohen's d_z is the standardized paired effect size (d_z = t/√5). n.s. = not significant.

Comparison	Mean ΔF1	SD ΔF1	95% CI	*t*	Cohen's d_Z	*P* value
vs. RETFound ViT-L	+0.0255	0.0059	[+0.0182, +0.0328]	9.70	4.34	< .001
vs. ViT-Base/16	+0.0075	0.0018	[+0.0052, +0.0098]	9.20	4.11	< .001
vs. Inception-V3	+0.0103	0.0028	[+0.0069, +0.0137]	8.38	3.75	< .001
vs. DenseNet-121	+0.0059	0.0059	[−0.0014, +0.0132]	2.24	1.00	.089 (n.s.)

Table S3. Corrected analysis: pairwise comparisons on the held-out internal test set. The first row is the single pre-specified primary contrast (uncorrected, α = .05); all others are Holm-corrected within their metric family of 36 pairs. Representative rows are shown. Of the 36 contrasts per family, 2 (macro-AUC), 3 (macro-F1) and 10 (accuracy) reached Holm-adjusted p < .05, and all of them are extreme versus extreme. Intervals are paired patient-level bootstrap throughout; p-values are paired bootstrap for macro-AUC and macro-F1 and McNemar for accuracy. No contrast involving hypertensive retinopathy is computed anywhere in this paper, and no architecture is declared superior.

Family	Contrast	Δ	95% CI	Test	*p* (Holm)
Primary (macro-AUC)	EfficientNet-B3 vs. ViT-Base/16	−0.0123	−0.0369 to +0.0142	Paired bootstrap	0.36*
macro-AUC (secondary)	ConvNeXt-Base vs. RETFound	+0.0610	+0.0320 to +0.0911	Paired bootstrap	0.036
macro-AUC (secondary)	ConvNeXt-Small vs. RETFound	+0.0559	+0.0239 to +0.0883	Paired bootstrap	0.036
macro-AUC (secondary)	EfficientNetV2-S vs. RETFound	+0.0492	+0.0176 to +0.0831	Paired bootstrap	0.099
macro-AUC (secondary)	All 8 adjacent-rank pairs	≤ 0.0230	All contain 0	Paired bootstrap	1.00
macro-F1	ConvNeXt-Base vs. RETFound	+0.0756	+0.0357 to +0.1191	Paired bootstrap	0.018
macro-F1	ConvNeXt-Base vs. Swin-Base	+0.0722	+0.0383 to +0.1078	Paired bootstrap	0.018

macro-F1	ConvNeXt-Small vs. Swin-Base	+0.0674	+0.0323 to +0.1046	Paired bootstrap	0.034
macro-F1	EfficientNet-B3 vs. ViT-Base/16	+0.0128	−0.0275 to +0.0551	Paired bootstrap	1.00
Accuracy	ConvNeXt-Base vs. RETFound	+0.0952	+0.0561 to +0.1341	McNemar	< 0.001
Accuracy	EfficientNet-B3 vs. RETFound	+0.0917	+0.0542 to +0.1298	McNemar	< 0.001
Accuracy	ConvNeXt-Base vs. Swin-Base	+0.0734	+0.0441 to +0.1023	McNemar	< 0.001
Accuracy	EfficientNet-B3 vs. ViT-Base/16	+0.0424	+0.0126 to +0.0716	McNemar	0.096

* Uncorrected by design: this is the sole primary test.

Table S4. Corrected analysis: ensemble operating characteristics on the held-out internal test set at the arg-max decision rule. **Post-hoc and descriptive; not pre-specified.** Sensitivity is per-class recall. Values are single-sample point estimates without resampling intervals, because no comparative claim rests on them. Predictive values depend on this cohort's prevalence and should not be read as screening performance. AMD = age-related macular degeneration; NPV = negative predictive value; PPV = positive predictive value.

**Class**	*n*	**Sensitivity (recall)**	**Specificity**	**PPV**	**NPV**
Normal	462	0.820	0.832	0.846	0.804
Diabetic retinopathy	234	0.803	0.950	0.855	0.929
Glaucoma†	43	0.698	0.961	0.484	0.984
Cataract†	42	0.905	0.983	0.731	0.995
Pathological myopia†	39	0.974	0.992	0.844	0.999
AMD†	37	0.676	0.988	0.714	0.986
Hypertensive retinopathy†	15	0.133	0.991	0.200	0.985
Any disease versus normal, seven classes collapsed:					
Any disease	410	0.832	0.820	0.804	0.846

n < 100; these rows are imprecise and no comparison is made between them. Any-disease AUC 0.891.

Table S5. Comparison with representative prior work on automated retinal disease classification. This table provides contextual positioning only; direct rank comparison is not appropriate owing to heterogeneity in datasets, class count, metrics, and validation methodology. AUC = area under the receiver operating characteristic curve; DR = diabetic retinopathy; F1 = support-weighted F1 score.

Study	Dataset	Task	Classes	Best reported metric
Gulshan et al, [1] 2016	Development set (128K)	DR detection	2	AUC 0.991
Abràmoff et al, [2] 2018	Primary-care trial (900 enrolled)	DR screening	2	Sensitivity 87.2%
Ting et al.,[3] 2017	Multi-ethnic (494K)	DR detection	2	Sensitivity 90.5%
De Fauw et al.,[4] 2018	NHS OCT (15K)	Referral triage	50+	Expert-level
Grassmann et al.,[5] 2018	AREDS (120K)	AMD severity	13	Accuracy 63.3% (Kω 0.92)
Zhou et al.[6] (RETFound), 2023	Multi-retinal	Multiple tasks	Various	Task-dependent
**This study**	**ODIR-5K+RFMiD+APTOS**	**7-class fundus**	**7**	**Acc 95.53%, F1 0.9507 (5-fold CV)**

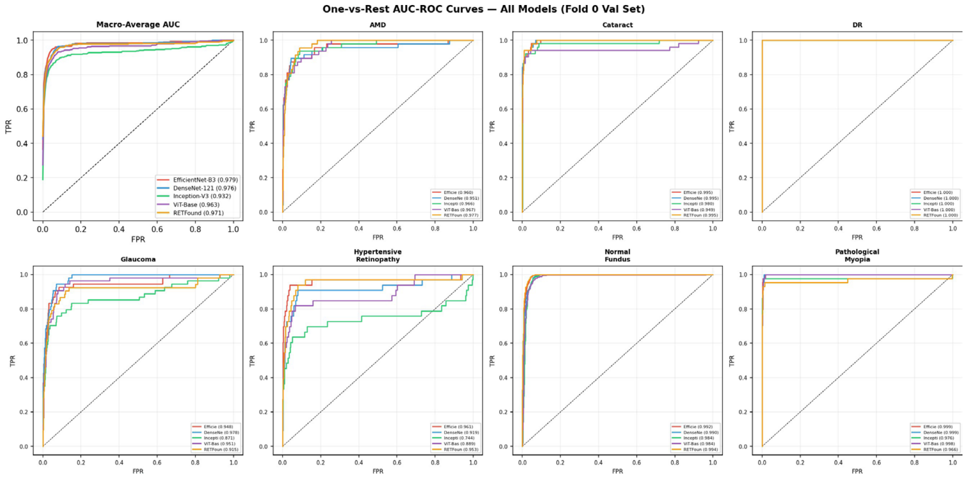

Figure S1. One-versus-rest AUC-ROC curves for all five architectures (fold 0 test set). Hypertensive retinopathy consistently shows the lowest AUC, reflecting funduscopic overlap with glaucoma and AMD. AMD = age-related macular degeneration; AUC = area under the receiver operating characteristic curve; DR = diabetic retinopathy.

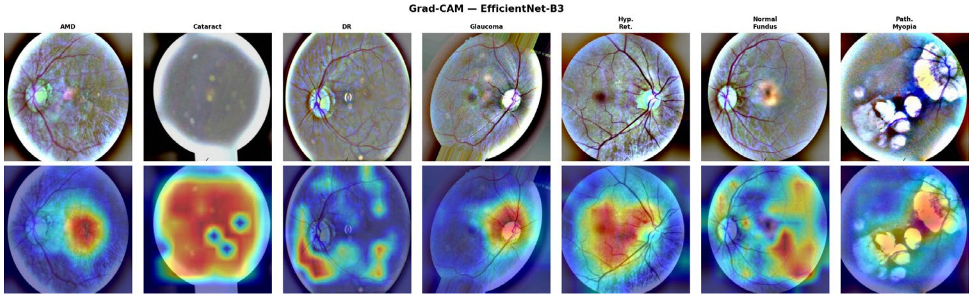

Figure S2. Grad-CAM activation maps for EfficientNet-B3 (fold 3 checkpoint), one image per disease class. Row 1: original fundus images. Row 2: Grad-CAM overlays. Checkmark = correct prediction; label = incorrect prediction. Activations localize to pathologically relevant structures in each class. AMD = age-related macular degeneration; DR = diabetic retinopathy.

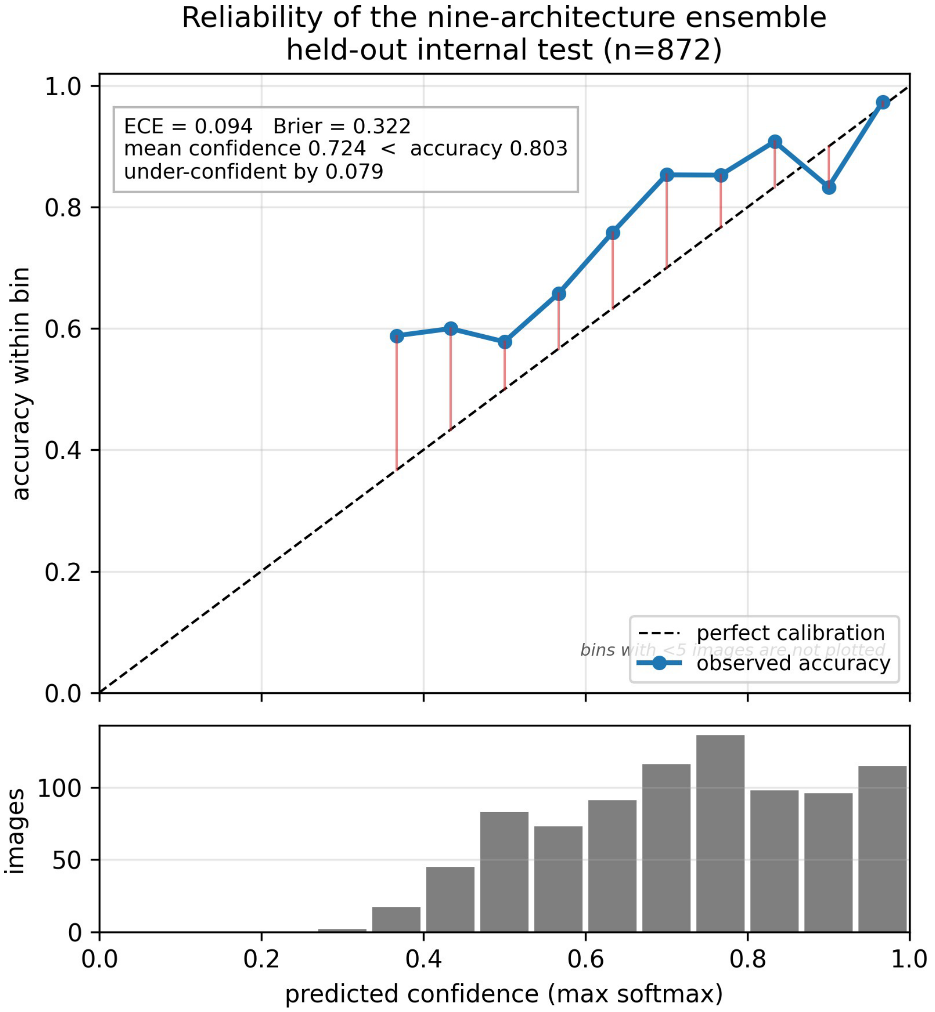

Figure S3. Corrected analysis: reliability diagram for the nine-architecture ensemble on the held-out internal test set (n = 872 eyes), with predicted confidence binned into 15 equal-width bins. **Post-hoc and descriptive; not pre-specified.** The curve lies above the diagonal across most of the confidence range: mean predicted confidence is 0.724 against an accuracy of 0.803, so the ensemble is under-confident by 0.079, with an expected calibration error of 0.094, a multi-class Brier score of 0.322 and a negative log-likelihood of 0.630. No temperature scaling or other recalibration was applied. The sparse bins are at the *low*-confidence end: bins above 0.7 are the most populated, at roughly 95–140 eyes each, while the lowest occupied bins hold 2, 17 and 45 eyes. Any bin containing fewer than five eyes is omitted from the plotted accuracy curve, but every bin, including the omitted ones, still contributes to the expected calibration error quoted above; here that rule drops one bin, at 0.27–0.33. No interval is plotted, and no claim in this paper rests on this figure.

References

1. Gulshan, V.; Peng, L.; Coram, M.; et al. Development and Validation of a Deep Learning Algorithm for Detection of Diabetic Retinopathy in Retinal Fundus Photographs. JAMA 2016, 316, 2402–2410.
2. Abramoff, M.D.; Lavin, P.T.; Birch, M.; Shah, N.; Folk, J.C. Pivotal Trial of an Autonomous AI-Based Diagnostic System for Detection of Diabetic Retinopathy in Primary Care Offices. NPJ Digital Medicine 2018, 1, 39.
3. Ting, D.S.W.; Cheung, C.Y.L.; Lim, G.; et al. Development and Validation of a Deep Learning System for Diabetic Retinopathy and Related Eye Diseases Using Retinal Images From Multiethnic Populations With Diabetes. JAMA 2017, 318, 2211–2223.
4. De Fauw, J.; Ledsam, J.R.; Romera-Paredes, B.; et al. Clinically Applicable Deep Learning for Diagnosis and Referral in Retinal Disease. Nature Medicine 2018, 24, 1342–1350.
5. Grassmann, F.; Mengelkamp, J.; Brandl, C.; et al. A Deep Learning Algorithm for Prediction of Age-Related Eye Disease Study Severity Scale for Age-Related Macular Degeneration From Color Fundus Photography. Ophthalmology 2018, 125, 1410–1420.
6. Zhou, Y.; Chia, M.A.; Wagner, S.K.; et al. A Foundation Model for Generalizable Disease Detection From Retinal Images. Nature 2023, 622, 156–163.
